# Ryanodine receptor 2–mediated calcium leak is associated with increased glyoxalase I in the aging brain

**DOI:** 10.1172/jci.insight.184041

**Published:** 2025-10-16

**Authors:** Elizabeth Woo, Dibyadeep Datta, Shveta Bathla, Hannah Beatty, Pinar Caglayan, Ashley Kristant Albizu, TuKiet T. Lam, Jean Kanyo, Mary Kate Joyce, Shannon Leslie, Stacy Uchendu, Jonathan DeLong, Qinyue Stacy Guan, Jiaxin Li, Efrat Abramson, Alison L. Herman, Dawson Cooper, Pawel Licznerski, Tamas L. Horvath, Elizabeth A. Jonas, Angus C. Nairn, Amy F.T. Arnsten, Lauren H. Sansing

**Affiliations:** 1 Interdepartmental Neuroscience Program, and; 2 Department of Psychiatry, Yale University School of Medicine, New Haven, Connecticut, USA.; 3 University of Colorado School of Medicine, Aurora, Colorado, USA.; 4 University of Rochester School of Medicine and Dentistry, Rochester, New York, USA.; 5 Department of Comparative Medicine,; 6 Keck MS & Proteomics Resource, and; 7 Department of Molecular Biophysics and Biochemistry, Yale University School of Medicine, New Haven, Connecticut, USA.; 8 Boston University, Boston, Massachusetts, USA.; 9 Johnson & Johnson Innovative Medicine, San Diego, California, USA.; 10 Departments of Neurology and Immunobiology, Yale University School of Medicine, New Haven, Connecticut, USA.; 11 Geisel School of Medicine at Dartmouth, Hanover, New Hampshire, USA.; 12 University of Michigan Medical School, Ann Arbor, Michigan, USA.; 13 University of Minnesota Medical School, Minneapolis, Minnesota, USA.; 14 Department of Internal Medicine, Section of Endocrinology, Yale University School of Medicine, New Haven, Connecticut, USA.; 15 Marine Biological Laboratory, Woods Hole, Massachusetts, USA.; 16 Department of Neuroscience, Yale University School of Medicine, New Haven, Connecticut, USA.

**Keywords:** Aging, Neuroscience, Alzheimer disease, Calcium, Proteomics

## Abstract

Alzheimer disease (AD) is characterized by plaques and tangles, including calcium dysregulation and glycated products produced by reactive carbonyl compounds. AD brains have increased glyoxalase I (GLO1), a major scavenger of inflammatory carbonyl compounds, at early, but not later, stages of disease. Calcium dysregulation includes calcium leak from phosphorylated ryanodine receptor 2 (pS2808-RyR2), seen in aged macaques and AD mouse models, but the downstream consequences of calcium leak remain unclear. Here, we show that chronic calcium leak is associated with increased GLO1 expression and activity. In macaques, we found age-related increases in GLO1 expression in the prefrontal cortex (PFC), correlating with pS2808-RyR2, and localized to dendrites and astrocytes. To examine the relationship between GLO1 and RyR2, we used S2808D-RyR2 mutant mice exhibiting chronic calcium leak through RyR2, and found increased GLO1 expression and activity in the PFC and hippocampus as early as 1 month and as late as 21 months of age, with a bell-shaped aging curve. These aged S2808D-RyR2 mice demonstrated impaired working memory. As with macaques, GLO1 was expressed in astrocytes and neurons. Proteomics data generated from S2808D-RyR2 synaptosomes confirmed GLO1 upregulation. Altogether, these data suggest potential association between GLO1 and chronic calcium leak, providing resilience in early stages of aging.

## Abstract 2

Chronic elevation of calcium levels in the brain is associated with an increase in glyoxalase I, an enzyme that protects cells from a toxic byproduct of glycolysis.

## Introduction

Late-onset Alzheimer disease (LOAD) is a devastating neurodegenerative disease that is the leading cause of dementia among the elderly. The etiology of LOAD remains elusive, making therapeutic development challenging. The disease consists of amyloid plaques and tau tangles, with synapse loss, neuroinflammation, mitochondrial defects, calcium dysregulation, and accumulation of advanced glycation end products (AGEs) (1–9). The pathology develops in a region-specific manner, especially targeting regions responsible for higher-order integrative processing, such as the entorhinal cortex (ERC), hippocampus, and the prefrontal cortex (PFC) (10).

AGEs are found in plaques, render tau resistant to proteolysis, and trigger activation of receptor for AGEs (RAGE), leading to neuroinflammation and suggesting a causal role in disease (4, 11). AGEs are produced by highly reactive carbonyl compounds, such as methylglyoxal (MGO), a by-product of glycolysis and gluconeogenesis, which can cause carbonyl stress (a type of oxidative stress) (12–14). The glyoxalase system, composed of glyoxalase 1 (GLO1) and 2 (GLO2), is primarily responsible for detoxifying MGO (15). In aging and LOAD in the human PFC, GLO1 exhibits age-related increases, followed by a reduction in the oldest individuals, suggesting a loss of resilience at the oldest ages (16, 17). Although GLO1 regulation remains unclear, its link to calcium was first described in the plant *Brassica juncea* (18).

Dysregulated calcium signaling in aging and LOAD has been well documented, including calcium leak from the smooth endoplasmic reticulum (SER) through ryanodine receptors, consistent with the extensive leak observed with presenilin mutations that cause early-onset disease (19–26). Calcium leak from the SER can be caused by hyperphosphorylation of the ryanodine receptor 2 (RyR2) channels at serine 2808 (S2808) by increased protein kinase A (PKA) signaling, both in the heart and brain (27–29). Phosphorylated RyR2 displaces calstabin2, which leads to increased calcium leak into the cytosol (30). Increased p-S2808-RyR2 is observed in postmortem human AD cortices and in aging macaque cortices, in both astrocytes and neurons, suggesting a fundamental role for RyR2-mediated calcium leak in age-related cognition loss (6, 31). Because cell culture paradigms utilize neurons at early developmental ages, they preclude the study of chronic processes and slow accumulating events that occur over extended time periods in long-lived neurons in situ. These possibly invisible events in vitro may be fundamental substrates in the progression of cognitive decline; thus, high-fidelity in vivo models are necessary. Rhesus macaques naturally develop p-S2808-RyR2 in the PFC and ERC, which correlates with the rise in tau hyperphosphorylation, as well as other signs of calcium dysregulation and mitochondrial dysfunction, amyloid plaques, synapse loss, and cognitive deficits (2, 7, 32–37). Importantly, perfusion fixation allows for high-resolution immuno-electron microscopy (immunoEM) to determine intracellular molecular expression in situ, which is not possible in humans. However, GLO1 expression and localization in the aging primate cortex are not well understood.

The current study aimed to investigate the association between GLO1 and chronic calcium leak through RyR2. We hypothesized that GLO1 expression may increase to counter the elevated cytosolic calcium levels. We used the macaque model to evaluate GLO1 changes with age and investigate its cellular and ultrastructural localization in layer III of the dorsolateral PFC (LIII dlPFC), the layer most associated with working memory operations and vulnerable to age-related pathology (38–40). To test the relationship between calcium leak and GLO1, we used a mouse model of S2808D-RyR2 that has a knockin mutation that mimics PKA phosphorylation of RyR2 at S2808, leading to increased calcium leak from SER through RyR2. Proteomics of synaptosomes from wild-type (WT) and S2808D-RyR2 mice identified neurodegeneration and metabolism pathways directly altered by chronic calcium dysregulation. We found elevated GLO1 expression and activity in the frontal cortex and hippocampus across age, which may be protective against the effects of calcium. Finally, we found significant functional consequences of chronic calcium leak, as aged S2808D-RyR2 mice demonstrated decreased working memory as compared with WT mice.

## Results

### GLO1 expression is increased with age in rhesus macaque dlPFC and correlates with levels of p-RyR2.

**Figure 1:**
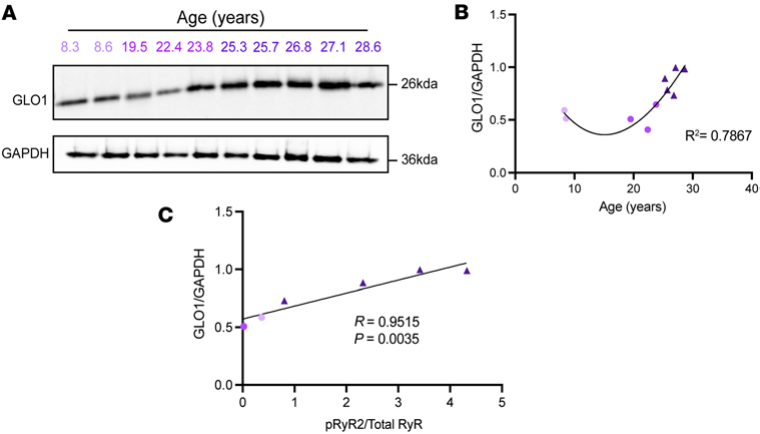
GLO1 expression increases with age and correlates with p-S2808-RyR2 in the rhesus macaque brain. (**A**) Immunoblot image of GLO1 and GAPDH in macaque cortical tissue across the age span (8.3–28.6 years). Age groups are color-coded (young, middle, aged). Numbers show the age of the macaque. (**B**) Age plotted against GLO1 expression with best fit regression cubic polynomial line; *R*^2^ is shown. (**C**) Quantification of p-S2808-RyR2 normalized to total RyR is plotted against GLO1 expression. A linear regression is shown, with the Pearson’s correlation coefficient and corresponding *P* value.

We immunoblotted for GLO1 across 10 monkeys from 8 years to 28.6 years (Figure 1A). From 10 to 20 years (middle age), the levels of GLO1 remained constant, followed by a dramatic increase in the older animals (Figure 1B). We did not observe a clear bell-shaped curve as observed in humans, where there is a loss of GLO1 at the oldest ages (16). It is possible that GLO1 would show decreases in macaques at still older ages, but this tissue is exceedingly rare. We observed a similar trend in the ERC, another region with early age–related pathology (Supplemental Figure 1).

To further characterize the relationship between altered RyR2 and GLO1, we examined the levels of p-RyR2 in a subset of monkeys and correlated it with their GLO1 expression levels. We hypothesized that there would be a positive correlation between the levels of p-RyR2 and GLO1, based on the potential role for GLO1 as a compensatory mechanism for calcium leak. We confirmed this positive correlation between the proportion of normalized p-RyR2 and normalized GLO1 (Figure 1C).

### GLO1 is highly expressed in dendrites and astrocytes in the aged macaque dlPFC.

**Figure 2:**
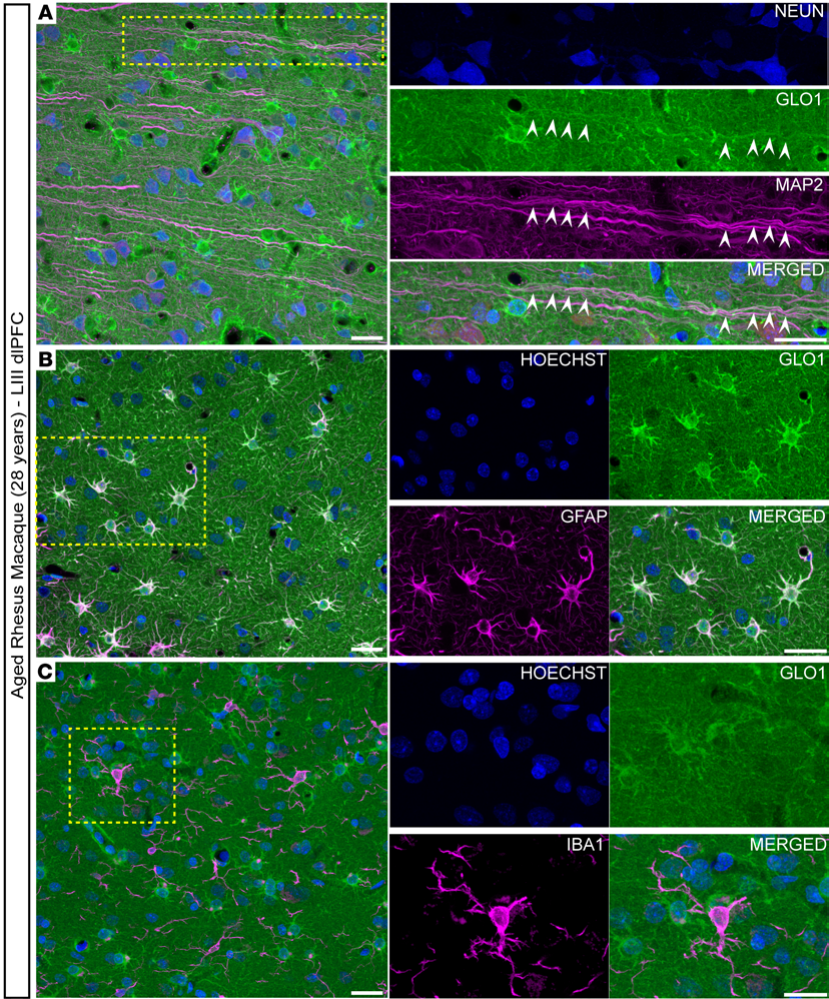
GLO1 cellular localization in the aged macaque LIII dlPFC. Representative confocal images of LIII dlPFC of an aged macaque (28 years) labeled with GLO1 (green) and (**A**) excitatory neuronal markers MAP2 (magenta) and NeuN (blue), (**B**) astrocytic marker GFAP (magenta) and nuclei marker (blue), and (**C**) microglial marker IBA1 (magenta) and nuclei marker (blue). The white arrowheads indicate colocalization of GLO1 within dendrites of excitatory neurons. Magnified images correspond to the dashed yellow boxes. Scale bars: 25 μm.

Transcriptionally, GLO1 is highly expressed by oligodendrocytes, astrocytes, and endothelial cells, and less abundantly expressed by neurons (41). Notably, GLO1 mRNA is minimally present in microglia (42). Given the increase in GLO1 with age, we examined its cellular expression patterns in LIII dlPFC using multiple-label immunofluorescence (MLIF) to differentiate pyramidal cells (MAP2), astrocytes (GFAP), and microglia (IBA1). We observed robust GLO1 expression in pyramidal cells, which was especially evident in dendrites (Figure 2A, white arrowheads, and Supplemental Figure 2A), but less visible in cell bodies. GLO1 was abundantly expressed in the soma of astrocytes and their processes (Figure 2B and Supplemental Figure 2B). We did not observe GLO1 colocalization with IBA1-expressing microglia in the aged macaque brain (Figure 2C and Supplemental Figure 2C).

### Ultrastructural localization of GLO1 in the aged macaque LIII dlPFC.

**Figure 3:**
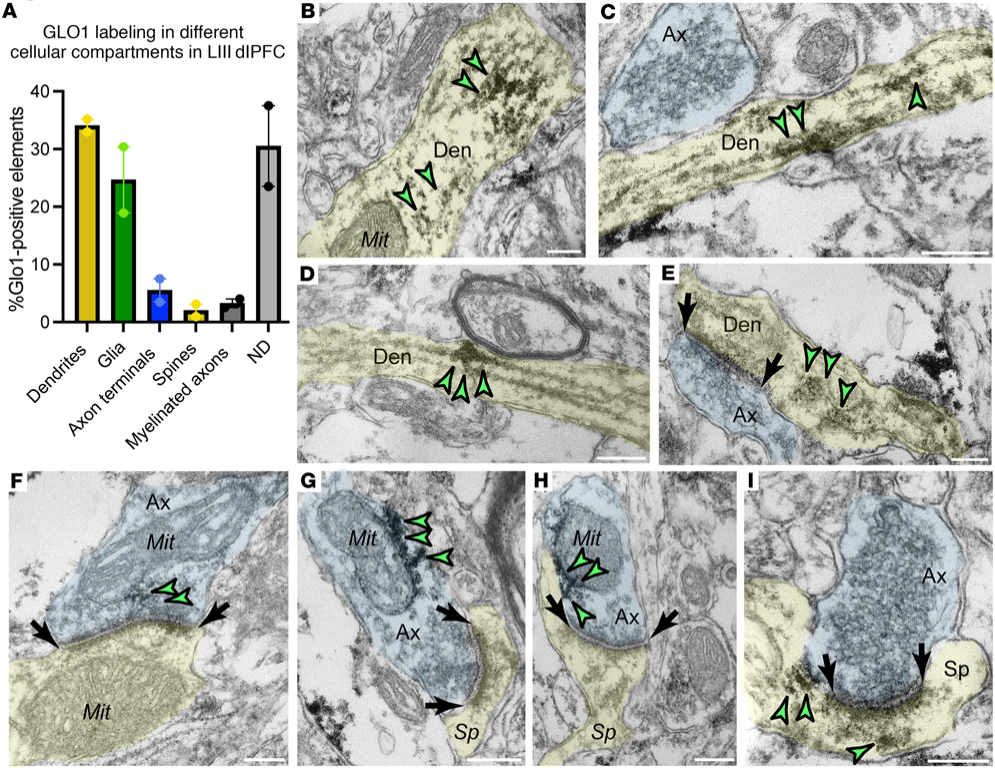
GLO1 immunoEM localization in neuronal compartments in LIII dlPFC of aged macaque. (**A**) Quantitative distribution of GLO1 across different cellular elements in aged macaque LIII dlPFC. Percentage of immunoperoxidase labeling (mean ± SEM) for GLO1 in glia, dendritic shafts, dendritic spines, axon terminals, and not determined structures (ND), *n* = 2 aged macaques. (**B**–**E**) GLO1 (green arrowheads) is observed along microtubule tracks within dendritic shafts. (**F**–**H**) GLO1 is also observed on mitochondria membranes in the presynaptic axon terminus as well as the perisynaptic membrane. (**I**) GLO1 labeling is also seen in the postsynaptic spine head and neck. Synapses are between black arrows. Profiles are pseudocolored for clarity and not all specific labeling is highlighted by arrowheads. Den, dendrites (yellow); Mit, mitochondria; Ax, axon (blue); Sp, spine (yellow). Scale bars: 500 nm.

ImmunoEM was used to determine the ultrastructural localization of GLO1 in the aged macaque LIII dlPFC. We analyzed 210 GLO1-immunopositive photomicrographs (total of 4144 GLO1 deposit sites). Consistent with MLIF data, the dendritic (approximately 34% of total sites) and glial compartments (approximately 24% of total sites) accounted for the majority of GLO1-positive sites, aside from the not determined (ND) category (approximately 37.5% of total sites) (Figure 3A). Further analyses of the micrographs showed that GLO1 expression was approximately 5.5% in presynaptic terminals and 2.0% within dendritic spines.

More in-depth analyses showed that the prominent postsynaptic localization of GLO1 in the dendritic shafts was targeted to microtubules (Figure 3, B–E, and Supplemental Figure 3, A and B) and the SER (Figure 3C). This finding was consistent with the correlation between p-S2808-RyR2 and GLO1 levels described above and the age-related expression of p-S2808-RyR2 in dendrites (Supplemental Figure 4, A and B). A closer examination of synapses showed GLO1 expression in both pre- and postsynaptic compartments (Figure 3, F–I). In glutamatergic-like axon terminals, we found GLO1 to be closely associated with the mitochondrial membrane as well as clustering around synaptic vesicles adjacent to the mitochondria (Figure 3, F–H, and Supplemental Figure 3B). We also observed GLO1 labeling within dendritic spines with potential localization near the SER spine apparatus (Figure 3I and Supplemental Figure 3C).

**Figure 4:**
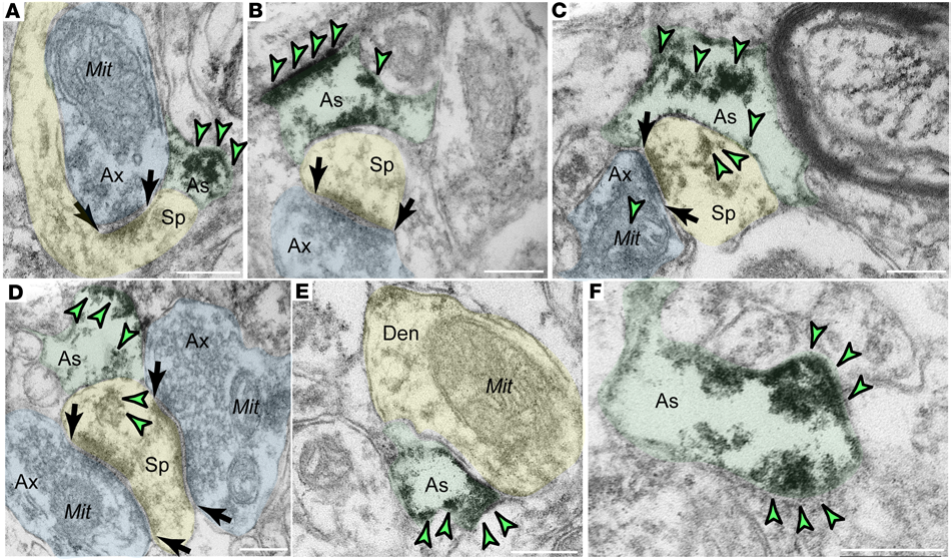
GLO1 immunoEM localization in glial processes in aged macaque LIII dlPFC. GLO1 immunoperoxidase labeling is abundant in glial leaflets within the neuropil. (**A**–**C**) GLO1 labeling can be seen filling the glial leaflet (**A**) as well as along the glial plasma membrane (**B**–**F**). GLO1 is observed in glial leaflets ensheathing glutamatergic-like synapses (**A**–**D**). Profiles are pseudocolored for clarity and not all specific labeling is highlighted by arrowheads. Den, dendrites (yellow); Mit, mitochondria; Ax, axon (blue); Sp, spine (yellow); As, astroglia (green). Scale bars: 500 nm.

GLO1 localization was also prominent in aged LIII dlPFC glial processes, likely astrocytic end feet (Figure 4 and Supplemental Figure 3, D–F). GLO1 was present within the intracellular space (Figure 4A and Supplemental Figure 3E), but was predominantly associated with the glial plasma membrane (Figure 4, B–F, and Supplemental Figure 3, D and F). We observed GLO1 labeling especially in glial leaflets ensheathing axospinous inputs, receiving glutamatergic-like asymmetric synapses (Figure 4, A–D). We observed similar patterns of expression of p-S2808-RyR2 in glia (Supplemental Figure 4, C–F).

In sum, the ultrastructural localization of GLO1 in the aged macaque dlPFC revealed a predominantly glial (likely astrocytic) and dendritic distribution (especially on microtubules), which is consistent with the MLIF data. The data also demonstrated robust labeling in axon terminals next to mitochondrial membranes and some labeling in postsynaptic spines.

### Proteomic profiling of S2808D-RyR2 synaptosomes from frontal cortex and hippocampus.

**Figure 5:**
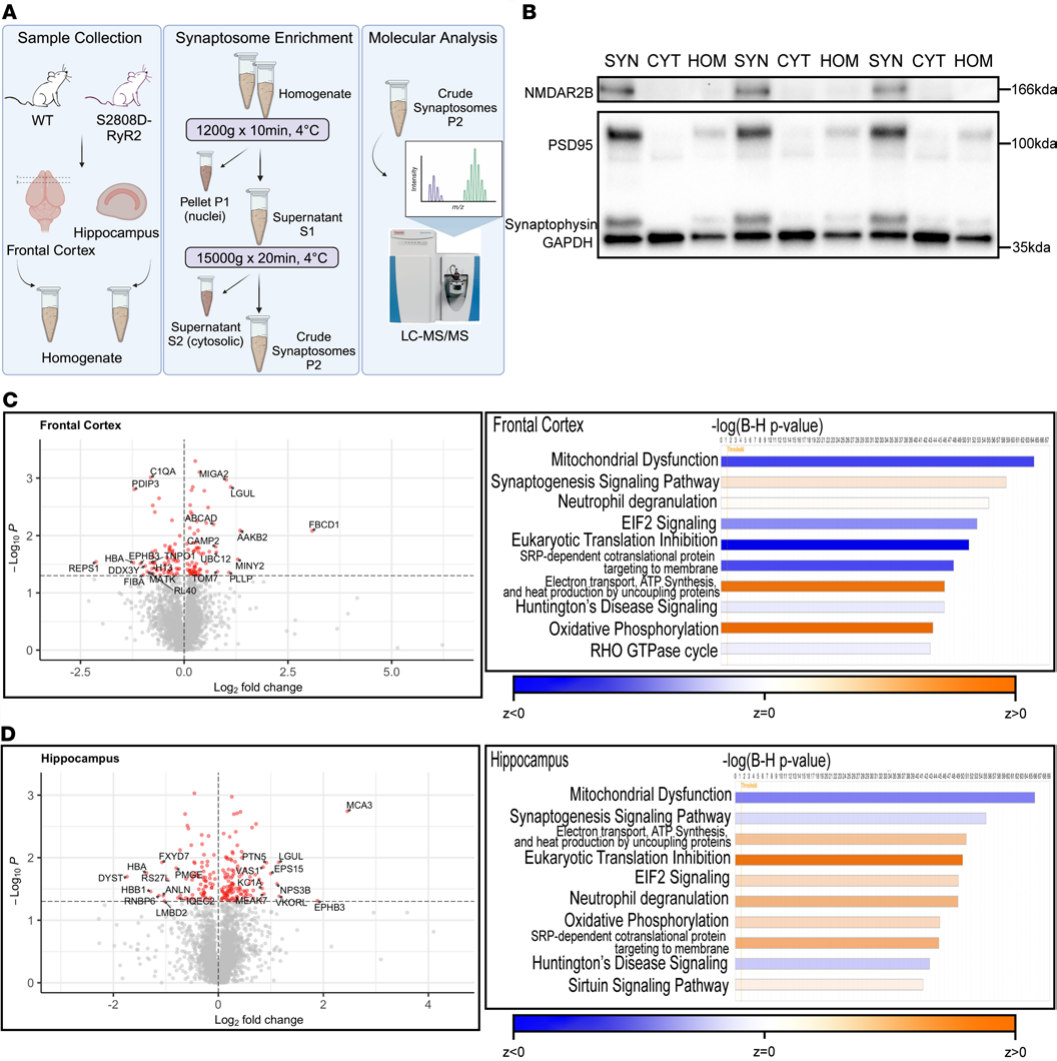
S2808D-RyR2–mediated calcium leak alters proteins in synaptosomes. (**A**) Schematic paradigm of experimental design. Synaptosomes from the frontal cortex and hippocampus were enriched from 3-month-old male WT and S2808D-RyR2 mice (*n* = 3 per group) and analyzed using LC-MS/MS. (**B**) Immunoblot image validating enrichment for synaptic proteins in the synaptosome suspension using Syn-PER reagent. NMDAR2B and PSD95 are postsynaptic proteins, while synaptophysin is a presynaptic protein. GAPDH is a cytosolic protein. (**C** and **D**) Volcano plots highlighting the proteins most enriched (red circles) in WT (negative log_2_[fold change]) and S2808D-RyR2 (positive log_2_[fold change]) synaptosomes in frontal cortex (**C**) and hippocampus (**D**) compared with WT. The horizontal dotted lines on the volcano plots signify an α of 1.3, or a *P* value of 0.05. The top 10 altered canonical pathways using the proteomic data sets as derived by Ingenuity Pathway Analysis (IPA) are represented by bar charts adjacent to each volcano plot. SYN, synaptosome; CYT, cytosol fraction; HOM, homogenate.

The macaque model demonstrated a correlative relationship between RyR2 and GLO1. Thus, we decided to examine the relationship by using a mouse model with genetically induced calcium leak through RyR2. The S2808D-RyR2 model replaces S2808 with an aspartic acid, leading to chronic calcium leak through the receptor from the SER. We harvested synaptosomes from the frontal cortex and hippocampus from 3-month-old male WT and S2808D-RyR2 mice to compare proteomic differences caused by chronic calcium leak, in an unbiased approach. Synaptic proteins were enriched from the brain tissue and processed for analysis by liquid chromatography–tandem mass spectrometry (LC-MS/MS) (Figure 5A). The enrichment of synaptic proteins of the synaptosomal fractions was verified using immunoblotting for specific synaptic proteins (e.g., PSD95, synaptophysin, NMDAR2B) (Figure 5B) and EM (Supplemental Figure 5). In both the hippocampus and frontal cortex samples, 3270 unique proteins were identified (where each identified protein had at least 1 unique peptide with a Mascot score of >35). Among these uniquely identified proteins, 190 and 132 were differentially abundant in S2808D-RyR2 versus WT synaptosomes in the hippocampus and frontal cortex, respectively (Figure 5, C and D, and Supplemental Dataset 1).

Many of the proteins that were more abundant in S2808D-RyR2 frontal cortex and hippocampus are implicated in mitochondria function and structure (e.g., MIGA2, TOMM7) (43–45). The most enriched protein in S2808D-RyR2 frontal cortex synaptosomes (8-fold higher than WT) was fibrinogen C domain–containing protein 1 (FIBCD1), which is a transmembrane receptor for chitin and glycosaminoglycans (46) (Table 1). In the hippocampus, the most enriched protein (5-fold higher than WT) was eukaryotic translation elongation factor 1 ε1 (MCA3), which is thought to respond to DNA damage (47, 48) (Table 1). GLO1, which is also known as lactoylglutathione lyase (LGUL), was among the most enriched proteins, and was higher (2-fold) in both frontal cortex and hippocampus of S2808D-RyR2 mice (Table 1). Among the proteins least abundant in S2808D-RyR2 mice compared with WT were hemoglobin subunits and complement protein C1QA (Supplemental Table 1).

Next, we conducted Ingenuity Pathway Analysis (IPA) to determine the most altered pathways. Both regions shared the top 10 most altered canonical pathways, suggesting that S2808-RyR2–mediated changes are targeting the same pathways in these 2 regions (Figure 5, C and D). IPA revealed changes in synaptogenesis signaling and mitochondrial dysfunction, as well as neurodegenerative disease pathways (Figure 5, C and D). Although most of the pathways were altered in the same direction in both regions, there were several that were altered in opposing directions, e.g., synaptogenesis and EIF2 signaling (Figure 5, C and D). One of the most upregulated pathways in both S2808D-RyR2 frontal cortex and hippocampus was oxidative phosphorylation, which is consistent with increased GLO1 expression (49). As expected, calcium signaling and calcium-related pathways were significantly upregulated in the S2808D-RyR2 mice compared with WT (Supplemental Table 2).

### GLO1 expression and activity are increased in 3-month-old and 12-month-old hippocampus and frontal cortex in the context of calcium dysregulation.

**Figure 6:**
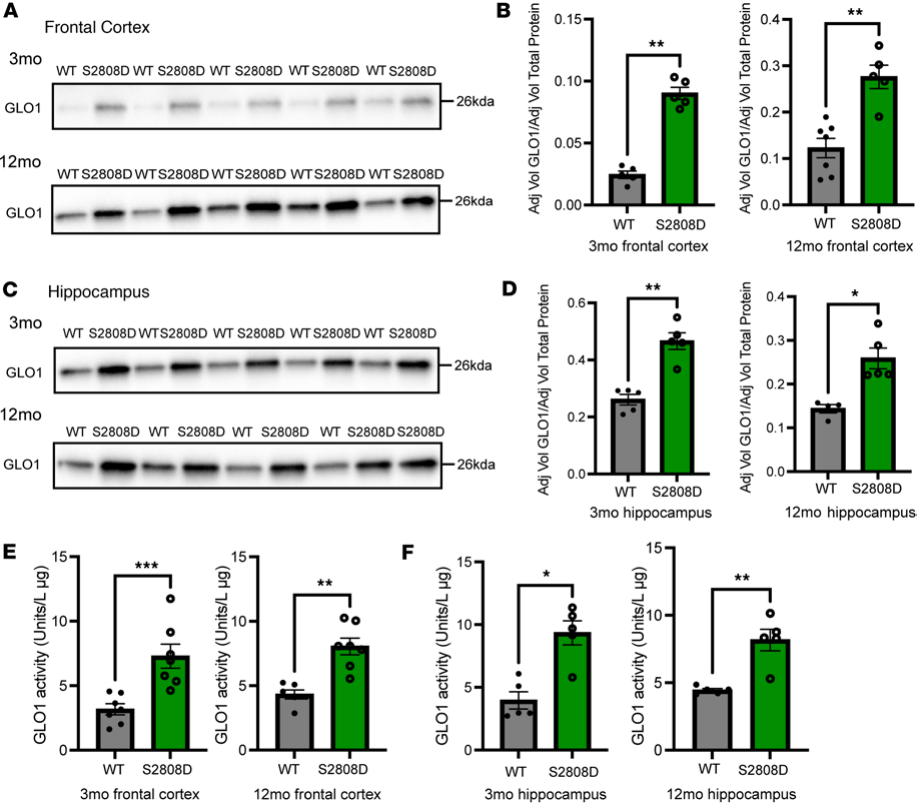
GLO1 expression and activity are increased in the frontal cortex and hippocampus in young and adult S2808D-RyR2 mice. (**A**) Immunoblot images of GLO1 expression in synaptosomes from 3-month-old and 12-month-old male S2808D-RyR2 frontal cortex. (**B**) Quantification of GLO1 expression normalized to total protein content based on stain-free blot (mean ± SEM). *n* = 5–7 per group per age. (**C**) Immunoblot images of GLO1 expression in synaptosomes from 3-month-old and 12-month-old S2808D-RyR2 hippocampus. (**D**) Quantification of GLO1 expression normalized to total protein content using stain-free blot (mean ± SEM). *n* = 5–7 per group per age. (**E**) Quantification of GLO1 activity levels (mean ± SEM). *n* = 5–7 per group per age. S2808D, S2808D-RyR2. *P < 0.05, **P < 0.01, ***P < 0.001 by Mann-Whitney *U* test.

To validate our proteomics findings, we immunoblotted for GLO1 from synaptosomes from the frontal cortex and hippocampus in both 3- and 12-month-old male mice (Figure 6, A–D). We found an increase compared with WT at both time points in the normalized expression of GLO1 to total protein in the S2808D-RyR2 synaptosomes from both the frontal cortex (Figure 6B) and hippocampus (Figure 6D). We found this increase in expression in female mice as well (Supplemental Figure 6). Furthermore, we observed this increase in GLO1 expression in S2808D-RyR2 synaptosomes as early as 1 month old and as late as 21 months old (Supplemental Figure 7). The increase was observed in total homogenate 3-month-old samples in hippocampus, suggesting multiple cell involvement (Supplemental Figure 8).

Next, we examined GLO1 activity in both regions at the 2 different ages, as GLO1 expression and activity may be differentially regulated (50–52). To do this, GLO1-mediated production of *S*-D-lactoylglutathione normalized to protein concentration was measured. At 3 and 12 months, there was higher GLO1 activity in the S2808D-RyR2 frontal cortex and hippocampus synaptosomes compared with WT (Figure 6, E and F).

These results indicate that calcium dysregulation through S2808-RyR2 is associated with both an increase in GLO1 expression and activity in synaptosomes across both regions in young and adult mice.

### GLO1 localization differs across cell types in the frontal cortex and hippocampus.

There are limited studies mapping the localization of GLO1 across different brain regions and cell types in mice. To investigate species differences in GLO1 localization and whether the increased expression in S2808D-RyR2 mice was driven by a specific cell type, we performed MLIF.

**Figure 7:**
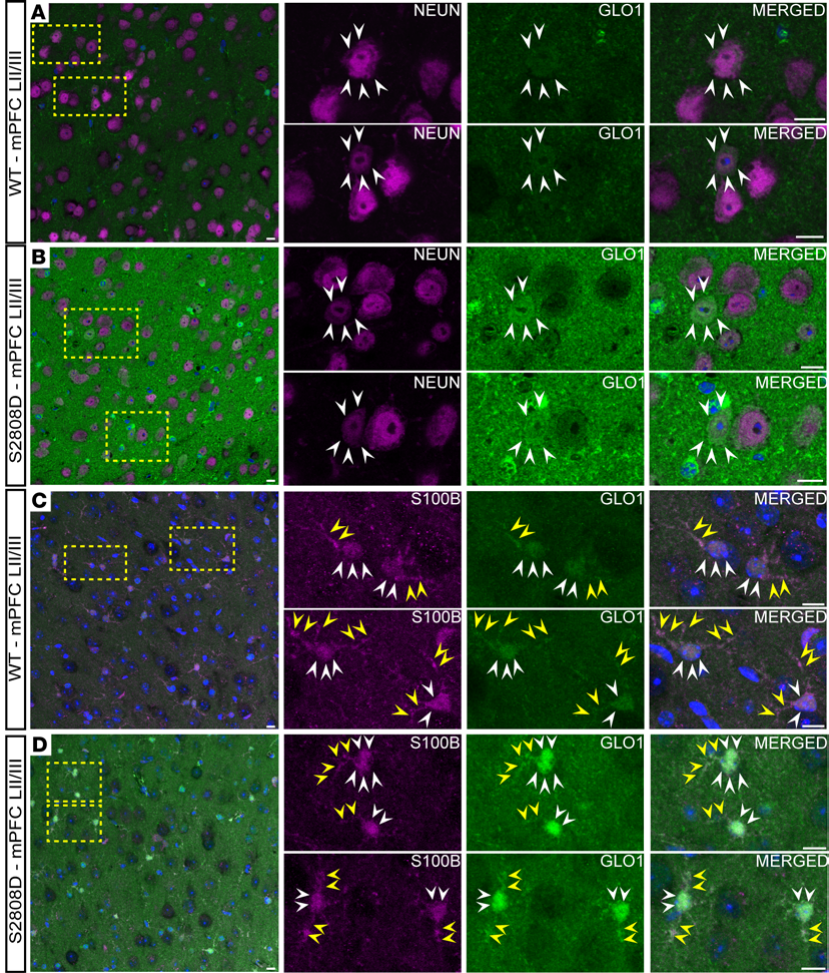
GLO1 localization in murine medial prefrontal cortex. Representative confocal images of layers II/III in the medial prefrontal cortex (mPFC LII/III) of 3-month-old male WT (**A** and **C**) and S2808D-RyR2 (**B** and **D**) mice. The magnified images correspond to the regions delineated by the yellow dashed boxes. (**A** and **B**) Neurons labeled with NeuN (magenta) colocalize with GLO1 (green), as identified by the white arrowheads. (**C** and **D**) Astrocytes labeled with S100 (magenta) colocalize with GLO1 (green). Processes of astrocytes are delineated by the yellow arrowheads, while the somas are indicated by the white arrowheads. S2808D, S2808D-RyR2. Scale bars: 10 μm.

We examined layer II/III of the medial PFC (LII/LIII mPFC) (Figure 7 and Supplemental Figure 9). We observed GLO1 localization to the soma of a subset of neurons (NeuN) in both WT (Figure 7A and Supplemental Figure 9A) and S2808D-RyR2 (Figure 7B, white arrowheads, and Supplemental Figure 9B) mPFC. Adjacent to GLO1-positive neurons, we also observed GLO1-negative neurons, suggesting a diversity in how neurons might respond to carbonyl stress. Given the robust neuropil GLO1 signal, it was challenging to appreciate clear proximal dendritic labeling, as seen in the macaque. In the S2808D-RyR2 cortex, we observed increased GLO1 intensity within the neuronal somas as compared with the somas in WT (Figure 7B and Supplemental Figure 9B).

We next examined colocalization of canonical astrocytic markers, S100 (Figure 7, C and D, and Supplemental Figure 9, C and D) and GFAP (Supplemental Figure 10), with GLO1. S100 was a better pan-astrocyte marker in LII/LIII mPFC than in the macaque dlPFC (53). Similar to the patterns observed in neurons, we observed GLO1 expression in the soma of astrocytes in WT (Figure 7C and Supplemental Figure 9C) and S2808D-RyR2 (Figure 7D and Supplemental Figure 9D). Furthermore, we saw robust GLO1 labeling of the fine astrocytic processes, as indicated by the yellow arrowheads. This was similar to what was observed in macaque dlPFC. We observed a qualitative increase in GLO1 intensity within the S2808D-RyR2 astrocytes, as seen in neurons, suggesting that astrocytes also likely contribute to the overall increase in GLO1 in synaptosomes, which capture astrocytic end feet. We also found GLO1 in oligodendrocytes or oligodendrocyte progenitor cells, as identified by Olig2 (Supplemental Figure 10, C and D). Interestingly, in both mouse and macaque PFC, there was no colocalization of GLO1 with IBA1-expressing microglia (Supplemental Figure 11, A and B).

**Figure 8:**
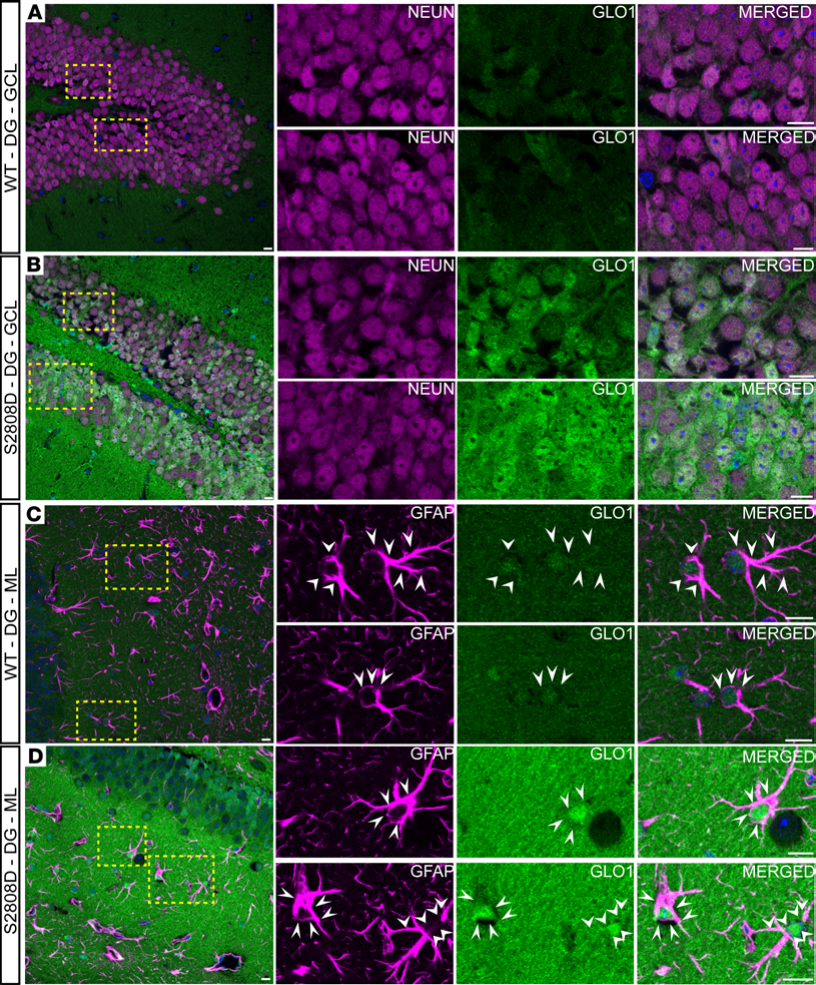
GLO1 localization in murine dentate gyrus. Representative confocal images of the dentate gyrus (DG) in 3-month-old male WT (**A** and **C**) and S2808D-RyR2 (**B** and **D**) mice. The magnified images correspond to the regions delineated by the yellow dashed boxes. (**A** and **B**) Neurons labeled with NeuN (magenta) colocalize with GLO1 (green) within the granule cell layer (GCL) of the DG. (**C** and **D**) Astrocytes labeled with GFAP (magenta) colocalize with GLO1 (green) within the molecular layer (ML), as identified by the white arrowheads. S2808D, S2808D-RyR2. Scale bars: 10 μm.

We next examined GLO1 expression in the dentate gyrus (DG) of the hippocampus (Figure 8 and Supplemental Figure 12). Overall, we observed similar patterns for GLO1 localization in the DG as observed in mPFC (Figure 8, A–D). GLO1 was moderately expressed by neurons in the granule cell layer (GCL) of the DG in WT (Figure 8A), and was more robustly expressed in the S2808D-RyR2 neurons in the same region (Figure 8B). Similarly, we found GLO1 colocalization with GFAP-positive astrocytes in the molecular layer (ML) (Figure 8, C and D) and with S100-positive astrocytes across both layers (Supplemental Figure 12, A and B). As in the mPFC, we also found GLO1 in oligodendrocytes and OPCs (Supplemental Figure 12, C and D), but not in microglia (Supplemental Figure 11, C and D).

Together the data provide evidence that, with the notable exception of microglia, several cell types contribute to the GLO1 increase observed in S2808D-RyR2 mice and that the mPFC and hippocampus share similarities in GLO1 cellular localization.

### GLO1 expression follows a bell-shaped curve with age in the S2808D-RyR2 frontal cortex.

**Figure 9:**
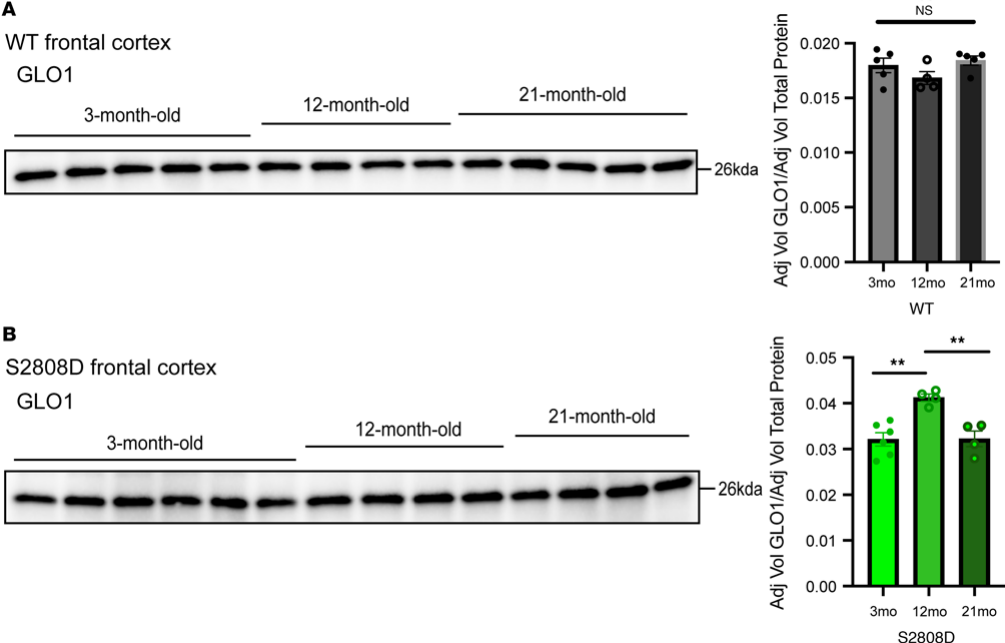
GLO1 expression follows a bell-shaped curve with age in S2808D-RyR2 frontal cortex. Immunoblot images of GLO1 expression in frontal cortex synaptosomes from male (**A**) WT and (**B**) S2808D-RyR2 mice at 3, 12, and 21 months old. Quantitative analysis was performed after normalizing to total protein content using stain-free blots (mean ± SEM). *n* = 5–6 per age per group. S2808D, S2808D-RyR2. **P < 0.005 by ordinary 1-way ANOVA with post hoc Tukey’s test.

Given the age-related changes in GLO1 expression in the human brain, we compared GLO1 expression across 3-, 12-, and 21-month-old frontal cortex synaptosomes. Consistent with the literature (54), we did not see age-related changes in WT mice (Figure 9A). In contrast, GLO1 expression was higher in 12-month-old S2808D-RyR2 frontal cortex synaptosomes than in both 3-month-old and 21-month-old synaptosomes, exhibiting a bell-shaped curve across the age span (Figure 9B).

In sum, we observe a similar bell-shaped curve in GLO1 expression in the S2808D-RyR2 frontal cortex, as seen in humans, but not in WT mice. However, we do not observe a difference in GLO1 expression between 21- and 3-month-old mice.

### Aged S2808D-RyR2 mice perform worse on delayed alternating task compared with WT mice.

**Figure 10:**
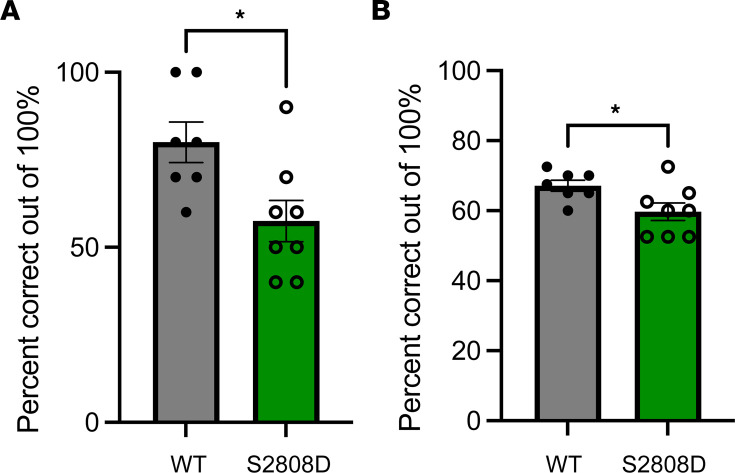
Aged S2808D-RyR2 mice perform worse on delayed alternating task compared with WT mice. Mice were trained on a spatial delayed alternation task in a T-maze, a test of working memory, behavioral inhibition, and attention regulation. After habituation (5 consecutive sessions), the last test session was analyzed for percentage correct (**A**) and the average of the last 4 test sessions (**B**). Results represent mean ± SEM percentage correct on the delayed alternation task. *n* = 7–8 per group. S2808D, S2808D-RyR2. *P < 0.05 by Mann-Whitney *U* test.

We hypothesized that increased calcium leak chronically through RyR2 would lead to functional deficits in the PFC of aged mice (at the end of the bell-shaped curve). In order to test this, we utilized a spatial delayed alternation task in a T-maze, which requires behavioral inhibition and attentional regulation. The mice were initially adapted to the maze, food rewards, and handling procedures to minimize the potential for contamination of affective influences on cognition. Once habituated, the mice were trained on the spatial delayed alternation task in the maze. We used mice at 21–22 months old, which was when we had observed a decrease in GLO1 expression (Figure 9B). We found that aged S2808D-RyR2 mice perform significantly worse than aged WT mice on the task with a delay (Figure 10). We observed this significant difference when examining the last test session (after 5 consecutive sessions for learning) (Figure 10A) as well as averaging the last 4 sessions (Figure 10B). These findings are consistent with prior data in aging macaques where chronic calcium leak leads to working memory deficits (6).

## Discussion

The current study examined the consequences of chronic calcium leak from the SER in the aging macaque and mouse brain. Previous research has shown evidence of calcium leak, tau pathology, and synapse loss in patients with LOAD and from aging macaques, but the direct downstream consequences of sustained calcium leak are poorly understood. We present that RyR2-mediated chronic calcium leak is associated with increased GLO1 expression and activity, as well as decreased working memory, and include a detailed cellular and ultrastructural mapping of GLO1 in the aged macaque dlPFC. The study also provides a synaptosome proteome data set from a chronic calcium dysregulation mouse model, which will be informative for future investigations on the downstream effects of elevated calcium in vulnerable areas of the brain.

There are potential limitations of the current study in the use of a constitutive knockin mouse model, S2808D-RyR2 (31). RyR2 is highly expressed in cardiomyocytes and there is evidence of increased calcium leak from the ER of S2808D-RyR2 cardiomyocytes (27). Although we cannot exclude the effects of cardiac dysfunction on the brain, we observed increased GLO1 expression in S2808D-RyR2 synaptosomes at 1 month of age, which precedes calstabin2 displacement of RyR2 in cardiomyocytes (27). Additionally, RyR2 is expressed by other neural cells, e.g., oligodendrocytes and astrocytes, potentially inducing calcium leak in these cells too. As a result, future studies using cell-type-specific mutations will be critical in dissecting the cell-type-specific chronic calcium leak effects. Finally, future studies utilizing specific RyR2 inhibitors will be important to testing the mechanisms underlying the relationship between GLO1 and RyR2.

GLO1 is a detoxifying enzyme that has been implicated in a number of diseases, such as mood disorders (55), autism (56, 57), anxiety (58, 59), schizophrenia (60, 61), diabetes (62, 63), cancer (64, 65), as well as aging (16, 66–69) and AD (17, 70–72). GLO1 in aging has been of particular interest, especially in light of a study which demonstrated that overexpression of GLO1 in *C*. *elegans* could extend their lifespan (66). Analyses of LOAD brain samples suggest that increases in GLO1 expression may provide resilience, as subcellular mapping of GLO1 expression with tau markers revealed an inverse relationship, where neurons that robustly expressed AT100 had weak expression of GLO1 (70). Studies have begun to elucidate the regulation of GLO1, but it remains poorly understood (73). The current finding of increased GLO1 expression and activity in the setting of chronic calcium leak suggests that this may be a resilience mechanism to counteract the toxic effects of sustained elevations in cytosolic calcium.

Few studies have mapped in great detail the localization of GLO1 at the protein level in different regions of the brain (16, 17, 74–76). In our study, we characterized the localization of GLO1 in cellular and subcellular compartments in the aged rhesus macaque and murine brain. Both macaque and mouse exhibited strong astrocytic and neuronal expression. However, there was much more obvious neuronal dendritic labeling in the macaque, as is described in human studies (70). The quantification of ultrastructural deposits in the aged macaque reflected our MLIF findings, where the abundance of GLO1 was highest in glia as well as dendrites. From the ultrastructural labeling, we observed delicate GLO1 labeling along the plasma membrane of glial leaflets, which are likely to be astrocytes. This would allow astrocytes to be able to immediately respond to any MGO spillover, as neurons are highly susceptible to MGO toxicity (77). This was a particularly notable finding, as existing single-cell nuclei sequencing data sets have shown that astrocytes have low levels of RyR2 mRNA. However, it is possible that this may be a technical limitation of single-cell nuclei sequencing methodology, as they lack astrocytic processes, or alternatively, there is a dissociation between transcription and protein levels. Recent proteomic studies that isolated subcellular compartments of astrocytes, specifically end feet and fine processes, demonstrated that the AQP4-labeled subcompartment had significantly elevated RyR2 levels as compared with all compartments (78). This provides additional protein evidence that RyR2 is expressed in astrocytic fine processes (which are important contact sites of synapses), supporting our ultrastructural findings. Future studies directly testing the role and mechanisms of RyR2-mediated calcium leak in astrocytes and comparing them to neuronal RyR2-mediated calcium leak would be of great importance.

In the dendrites, we found GLO1 to associate directly with microtubules. The link between microtubules and glyoxalases has been hypothesized; therefore, the physical association suggests GLO1 trafficking from the soma to distal dendrites along the microtubules (79). In the postsynaptic spine, we observed GLO1 close to the postsynaptic density as well as in association with the SER, where RyR2s are located and calcium leak occurs. In axon terminals, GLO1 proximity to mitochondria may facilitate *S*-D-lactoylglutathione entry into the mitochondria and supplement glutathione, a critical antioxidant (80). These findings elucidate the precise localization of GLO1 in cellular subcompartments in the rhesus macaque dlPFC, building upon earlier findings from humans and providing insights into GLO1 biology.

The cellular localization of GLO1 in the mouse was generally similar to that in the macaque, with astrocytes robustly expressing GLO1, as well as neurons and oligodendrocytes to a lesser extent in both the hippocampus and frontal cortex of WT and S2808D-RyR2 mice. The increase in GLO1 in S2808D-RyR2 seems to be due to an overall increase across all these cell types, rather than by a single cell type. However, the differences in GLO1 levels across cell types likely lead to variations in their abilities to respond to glycolytic flux, energy requirements, and MGO production. For instance, in vitro, astrocytes have a more efficient glyoxalase system than neurons, reflected in the higher GLO1 expression and activity (77). Interestingly, we did find that the DG neurons robustly expressed GLO1 within the soma and dendrites. This could be due to the fact that in the murine brain, the neurons in the GCL undergo neurogenesis well into adulthood, which could lead to a higher burden of GLO1 substrates, as there is increased metabolism (81), thus rendering them vulnerable when there is loss of GLO1. Another potential reason could be due to the lack of astrocytic leaflets ensheathing neurons in the GCL, which means the neurons cannot rely on neighboring glia for their GLO1 to quickly metabolize MGO spillover (81).

Regional differences and similarities between the hippocampus and frontal cortex were reflected in the IPA analysis. Although most of the pathways highly altered by chronic RyR2 signaling were found in both the hippocampus and frontal cortex, the pathways were often altered in opposing directions, consistent with their differing functions (working vs. long-term memory) and molecular regulation (high levels of cAMP/PKA/calcium signaling harmful vs. beneficial, respectively). For instance, EIF2 signaling was downregulated in S2808D-RyR2 frontal cortex, but upregulated in S2808D-RyR2 hippocampus. This could reflect differences in calcium handling capacities between the 2 regions (e.g., expression of calcium binding proteins), and as a result, could help explain regional vulnerabilities (82). For example, pathology occurs earlier in the hippocampus than in the frontal cortex in human LOAD and we observed potentially more inflammatory processes in the S2808D-RyR2 hippocampus as compared with the frontal cortex: upregulated EIF2 signaling and downregulated synaptogenesis signaling. Furthermore, some of the proteins that we observe to be reduced in S2808D-RyR2 frontal cortex have been implicated in neuroinflammation, including complement proteins, e.g., C1QA, suggesting a more compensated stage. The differences in pathway regulation could also reflect the differences in RyR2 expression. For instance, RyR2 is robustly expressed in the cortex and DG, although less so in CA1–CA3 (83). Our proteomics data support regional differences in calcium handling, which we propose contributes to the regional differences in vulnerability to calcium-mediated neurodegeneration. In contrast, the oxidative phosphorylation pathway was significantly upregulated in both regions, based on increases in electron transport chain–related proteins, e.g., ATP synthase, complex I. This is consistent with prior work using a GLO1-knockout model, suggesting a consistent role of GLO1 in mediating bioenergetics across different brain regions (49). However, determining whether this leads to increased ATP synthesis or functional changes to the mitochondria will require further investigation.

Studies of changes in GLO1 expression across the age span have often demonstrated a bell-shaped curve, e.g., in murine liver and spleen, with an increase up to 12–14 months, followed by a decline at 24 months. This loss of GLO1 at old age may represent a loss of compensation and vulnerability to degeneration. Importantly, there is some organ and species specificity, as GLO1 expression in WT murine brains does not follow a bell-shaped curve (54). In humans, GLO1 expression in the brain increases with age, up until 55 years, followed by a steady decline in aged individuals (80 years) (16). Similarly, there was increased GLO1 expression in Brodmann area 22 of early (Braak) stage LOAD and lower in later stages (17). We hypothesized that GLO1 expression would follow a similar bell-shaped curve in our macaques. We did find a steep increase with age, but levels plateaued in the 2 oldest monkeys. It is possible that still older animals may have shown declines, but macaques of such ages are exceedingly rare. A bell-shaped curve was seen with age in the S2808D-RyR2 mice, suggesting that a compensatory response may be overwhelmed at oldest ages.

Chronic calcium leak has been linked to cognitive impairment (6, 31). In aging macaques, prior studies have demonstrated evidence of increased calcium dysregulation and deficits in task-related neuronal firing and cognitive performance, which can be normalized pharmacologically by reducing calcium leak (6). Thus, we hypothesized that S2808D-RyR2 mice at the end of the bell-shaped curve, which have had chronic calcium exposure and a relative decrease in GLO1 expression, would have decreased working memory. We found that compared with their WT counterparts, the S2808D-RyR2 mice performed worse on the delayed alternating task. This aged S2808D-RyR2 mouse serves as a potentially relevant model for testing novel therapeutics and further elucidating the mechanism by which RyR2 signaling directly or indirectly impacts GLO1 expression. For instance, we can use GLO1 agonists (including upstream modulators of GLO1, like NRF2) to test whether working memory can be rescued in these mice or even administered prophylactically. One recent study demonstrated increasing neuronal GLO1 expression rescued AGE-induced deficits in synaptic plasticity and transmission, as well as learning and memory (84). Interestingly, GLO1 has also been of great interest as a therapeutic target in multiple solid cancers. There have been studies examining GLO1 agonism as a way to prevent procancer effects. For instance, in a mouse xenograft model, GLO1-depleted breast cancers showed increased tumorigenic and metastatic potential and similarly, increased aggressiveness of colorectal cancer patients’ cancer cells inversely correlated with GLO1 activity (85, 86). These may have additional brain-protective benefits such as improved cognition. At the same time, GLO1 inhibitors, such as *S*-*p*-bromobenzylglutathione cyclopentyl diester, are being investigated as chemotherapy adjuncts to target the highly metabolically active GLO1-expressing cancer cells (64). Our results suggest that screening for adverse cognitive changes in patients receiving brain-penetrant GLO1 inhibitors will be critical, as there may be the possibility of cognitive impairment.

Altogether, our results reveal an association between chronic calcium leak and GLO1 in the murine and macaque brain. We found an increase in GLO1 in our aging macaque model and in the S2808D-RyR2 murine model of chronic calcium leak. Furthermore, we characterized the localization of GLO1 in both models and generated a murine synaptosome proteomics data set across 2 brain regions, which provide insights into GLO1 biology. Lastly, we demonstrated working memory deficits in aged S2808D-RyR2 mice as compared with aged WT mice. Future investigations are necessary to map out the precise kinetics, regulation, and role of GLO1 in disease contexts to be able to develop strategies that maximize the benefits of GLO1.

## Methods

### Sex as a biological variable.

Both male and female mice were examined.

### Mice.

Male and female mice (1, 3, 12, 21, 22 months) were used. C57BL/6J (WT) were obtained from The Jackson Laboratory. S2808D-RyR2 mice were obtained from Andrew Marks laboratory (Columbia University, New York, New York, USA). All mice were bred under specific pathogen–free conditions with a 12-hour light/dark cycle in a temperature-controlled environment and ad libitum access to water and food pellets.

### Rhesus macaque brain tissue.

Two rhesus macaque brains were utilized for MLIF and immunoEM, one from a 28-year-old male (28y M), and one from a 30-year-old female (30y F). Brains were sectioned coronally at 60 μm on a vibratome (Leica) across the entire rostrocaudal extent of the dlPFC. The sections were cryoprotected using increasing concentrations of sucrose solution (10%, 20%, and 30% each overnight), cooled using liquid nitrogen, and stored at –80°C. To enable penetration of immunoreagents, all sections went through 3 freeze-thaw cycles in liquid nitrogen. Five aged (28.6y M, 27.1y F, 26.8y F, 25.7y F, 25.3y F), 3 middle-aged (23.8y M, 22.4y F, 19.5y F), and 2 young (8.6y F, 8.3y F) for immunoblotting. The subjects had varied medical and health histories. Postmortem intervals were minimized to the greatest extent possible.

### Antibodies.

Primary antibodies against the following proteins were used in this study: GLO1 (Abcam, ab81461; 1:500, used for mouse and macaque immunofluorescence), MAP2 (Abcam, ab5392; 1:1000), NeuN (Millipore, ABN78; 1:1000), GFAP (Neuromab, N206A/8; 1:1000), OLIG2 (Cell Signaling Technology, 65915; 1:200), S100 (R&D Systems, AF1820; 1:100), GLO1 (Abcam, ab137098; 1:1000, used for mouse Western blots), synaptophysin (Abcam, ab14692; 1:200), PSD95 (Neuromab, K28/43; 1:1000), IBA1 (Cell Signaling Technology, 17198T; 1:200), IBA1 (Synaptic Systems, 234 009; 1:1000), GAPDH (Millipore, aB2302; 1:10,000, mouse Western blots), GAPDH (Millipore, CB1001-500; 1:10,000, macaque Western blots), and GLO1 (Thermo Fisher Scientific, MA1-13029; 1:1000, used for macaque Western blots). All secondary antibodies used were conjugated with Alexa dyes from Thermo Fisher Scientific (1:250). HRP-conjugated antibodies were from Jackson ImmunoResearch (1:10,000).

### GLO1 activity assay.

GLO1 activity was assessed using the Glyoxalase I Activity Assay Kit (Sigma-Aldrich, MAK114), following the manufacturer’s instructions. Briefly, the lysates were thawed from –80°C. Total protein was quantified by the Pierce BCA Protein Assay Kit (Thermo Fisher Scientific, 23225). Samples were diluted to 0.6–0.7 μg/μL and equal amounts of protein were separated into the sample and blank tubes. Master Reaction Mix was added first to the sample tubes and incubated for 20 minutes at room temperature (RT), followed by 4 M perchloric acid incubation on ice for 15 minutes. The samples were centrifuged for 5 minutes at 20,800*g* at 4°C. The cleared supernatant was plated on a 96-well UV multiwell plate. Perchloric acid (4 M) was added to the sample blank tubes for 15 minutes on ice followed by another 15-minute incubation with the Master Reaction Mix. The sample blank tubes were centrifuged and supernatants transferred to wells. The samples’ absorbances were read at 240 nm using the Infinite M Plex Plate Reader (Tecan).

### GLO1 activity assay analysis.

GLO1 activity was calculated using the equation, activity =175 × (A240 sample − A240 blank) × 1.35. The activity was normalized to protein concentration determined by BCA.

### Synaptosome preparation.

Mice were deeply anesthetized with 1%–3% isoflurane inhalation and ventilating with oxygen-enriched air (20%:80%). All solutions were ice-cold. They were perfused using 20 mL ice-cold PBS, followed by decapitation. The brains were isolated and placed on an ice-cold petri dish. Using the Allen atlas to determine the regions of interest, the prefrontal cortex region and hippocampus were dissected, pooled from both hemispheres, and placed into preweighed 1.5 mL Eppendorf tubes containing 1 mL Syn-Per Synaptic Protein Extraction Reagent (Thermo Fisher Scientific, 87793) supplemented with 1× cOmplete, Mini, EDTA-free Protease Inhibitor Cocktail (Sigma-Aldrich, 11836170001) and 1× PhosSTOP (Sigma-Aldrich, 4906837001). Synaptosome enrichment was accomplished following the manufacturer’s recommendations. The tissue was homogenized in with 10–12 slow strokes in a 1 mL Dounce-type glass homogenizer (VWR, 357538) on ice. The homogenate was centrifuged at 1200*g* for 10 minutes at 4°C and the supernatant was further centrifuged at 15,000*g* for 20 minutes at 4°C. The pellet was then resuspended in RIPA buffer (Thermo Fisher Scientific, 89900) supplemented with protease and phosphatase inhibitor cocktails (same as above) to obtain concentrations of 3–4 μg/μL. The synaptosome isolates were stored at –80°C.

### Total homogenate preparation.

Murine PFC and hippocampus were isolated and homogenized by Dounce as described above for synaptosome preparation, with the modification that tissue was homogenized in 1 mL RIPA buffer supplemented with 1× protease and phosphatase inhibitor cocktails to a final concentration of 800 μL per 100 mg. The tubes were left on ice for 30 minutes and vortexed every 10 minutes for 20 seconds. The homogenate was centrifuged at 20,800*g* at 4°C for 15 minutes and the supernatant was stored at –80°C.

### MS proteomics.

The synaptosome samples (see *Synaptosome preparation* above, left in Syn-Per) were vortexed for 3 minutes and then sonicated in a water bath at 37°C for 30 minutes. The lysates were centrifuged at 14,600*g* at 4°C for 10 minutes to pull debris down. An aliquot of 65 μg was transferred into a new tube and the volume was brought up to 50 μL with water. Proteins were reduced with 5 μL of 45 mM dithiothreitol at 37°C for 30 minutes, and subsequently alkylated with 5 μL of 100 mM iodoacetamide at RT for 30 minutes in the dark. The proteins were then precipitated twice by acetone precipitation. The resulting protein pellet was dissolved in 50 μL of 8 M urea/0.4 M ammonium bicarbonate, diluted with 148 μL water, and digested with 1 μL of 0.5 μg/μL trypsin at 37°C for 4 hours followed by an additional 1 μL of 0.5 μg/μL trypsin at 37°C, overnight. The digestion was quenched by acidifying with 10 μL 20% trifluoro acetic acid. The peptide solution was then desalted using a macro RP C18 desalting columns (The Nest Group). Eluted peptides were dried in a SpeedVac and stored at –80°C until data collection. LC-MS/MS label-free quantitative analyses were carried out on a Q-Exactive Plus mass spectrometer coupled to a Waters Symmetry C18 180 μm × 20 mm trap column and a 1.7 μm, 75 μm × 250 mm NanoACQUITY UPLC column (35°C). Additional details on UPLC and mass spectrometer conditions can be found in Charkoftaki et al. (87). The LC-MS/MS data were processed using Progenesis QI Proteomics software (Nonlinear Dynamics, version 4.0), and protein identification was carried out using the Mascot search algorithm (Matrix Science). For all analyses, the list of proteins identified using the stringent criteria (95% confidence in ID) for quantification of proteins (requiring at least 1 unique peptide per protein with Mascot score of >35) was used. All protein IDs had an FDR of 2% or less.

### Immunoblotting and analysis of murine tissue.

Lysates were thawed on ice and total protein was quantified by BCA. Equal amounts of protein (6–12 μg per sample) were loaded onto 4%–15% mini-PROTEAN TGX stain-free gels (Bio-Rad, 4568085) and separated at 150 V for 1 hour. The gels were activated using the stain-free gel option on the ChemiDoc XRS+ Imaging System (Bio-Rad, 1708265). Proteins were transferred onto a 0.45 μm Immun-Blot Low Fluorescence PVDF membrane (Bio-Rad, 1620264) using the TransBlot Turbo Transfer System (Bio-Rad, 1704150), and the membrane was reimaged on the ChemiDoc using the stain-free membrane setting to capture total protein in each lane. Membranes were blocked with 5% BSA (Sigma-Aldrich, A3294) in 20 mM Tris-HCl, 140 mM NaCl, 0.05% Tween 20, pH 7.5 (1× TBS-T) and probed with primary antibodies overnight at 4°C. The membranes were washed for 45 minutes in 1× TBS-T at RT and incubated with the appropriate HRP-linked secondary for 1 hour at RT. Membranes were washed in 1× TBS-T for 20 minutes. Membranes were developed with Clarity Western ECL Substrate (Bio-Rad, 1705060) according to the manufacturer’s instructions. Chemiluminescent and colorimetric images were acquired using the ChemiDoc. Densitometric analysis was done on the blots using Image Lab software (Bio-Rad). All quantification was normalized to the total protein content as measured on the stain-free blot.

### Immunoblotting and analysis of macaque tissue.

Brain tissue (dIPFC, ERC; 100 mg) was lysed in lysis buffer (200 mM NaCl, 10 mM HEPES, 10 mM EGTA, 10 mM EDTA, 1% Triton X-100, phosSTOP phosphatase inhibitor, and cOmplete Mini protease inhibitor) with 20 strokes in a homogenizer. Cell debris was removed by centrifugation for 15 minutes (13,000*g*) at 4°C. The supernatant was collected, and protein concentration was determined with Bradford Assay (Bio-Rad). Protein (40 μg per lane) was boiled for 5 minutes at 100°C in SDS-loading buffer with DTT. The samples were separated in 4%–20% Tris-glycine gels using 150 V over 1.5 hours in a Criterion cell (Bio-Rad). Proteins were transferred onto a 0.45 μm nitrocellulose membrane at 300 mA for 1.5 hours in a Criterion blotter (Bio-Rad). After 1 hour blocking at RT in TBS-T containing 3% BSA, the membrane was probed overnight with anti-GLO1 (Thermo Fisher Scientific, MA1-13029; 1:1000) or anti-GAPDH (Millipore, CB1001-500; 1:10,000) antibodies diluted in TBS-T containing 3% BSA at 4°C. Membranes were washed 3 times with TBS-T (removal of unbound antibody) and incubated with fluorescent secondary antibodies (1:10,000) of the appropriate species for 1 hour at RT. Blots were rinsed with Milli-Q water and analyzed using a LI-COR Odyssey scanner. Image Studio Lite (LI-COR) was used for band quantification and background subtraction was done prior to quantification. The expression of targets normalized calculating the ratio of the target band intensity to GAPDH band intensity. Normalized data were plotted in Prism software and the Mann-Whitney *U* test was performed on the grouped analysis.

### Immunofluorescent staining of mouse tissue.

Mice were deeply anesthetized as described above and then perfused using 20 mL ice-cold PBS followed by 15 mL of ice-cold 4% paraformaldehyde (PFA) (Electron Microscopy Sciences, 15714) in PBS. The whole brains were dissected and placed into glass vials with 4% PFA and fixed overnight at 4°C. The brains were sectioned coronally at 30 μm on a vibratome (Leica) from the olfactory bulbs to the visual cortex. The sections were stored in 1× TBS with 0.05% sodium azide until use. Antigen retrieval was performed using Antigen Unmasking Solution (Vector Labs, H-3300-250) in a steam bath for 15 minutes. The tissue was cooled on the bench top for 10 minutes, followed by 2–3 washes with deionized water. The sections were washed for 10 minutes in 1× TBS before blocking for 1 hour at RT in 1× TBS containing 5% BSA, 2% Triton X-100, and 10% normal goat serum. Sections were incubated for 48 hours at 4°C with specific primary antibodies in dilution buffer (2% BSA, 2% Triton X-100, and 1% normal goat serum). Appropriate secondary antibodies in dilution buffer were used at 1:250 dilution for 3 hours at RT. All subsequent steps were performed in the dark and at RT. The sections were washed for 1 hour in 1× TBS and counterstained with Hoechst 33342 for 10 minutes (1:10,000; Thermo Fisher Scientific, H3570). The sections were washed for 30 minutes in 1× TBS before mounting onto slides using ProLong Gold Antifade Mountant (Invitrogen, P36930).

### Immunofluorescent staining of macaque tissue.

Immunofluorescent staining was carried out on free-floating sections from macaques. The sections were washed in 1× TBS for 1 hour at RT. Antigen retrieval was performed using Antigen Unmasking Solution in a steam bath for 25 minutes. The tissue was cooled on the bench top for 10 minutes, followed by several washes using deionized water. To reduce background autofluorescence, the sections were immersed in 0.5% sodium borohydride in 1× TBS for 10 minutes at RT, followed by washing in 1× TBS for 1 hour at RT (88). The sections were blocked for 1 hour at RT in 1× TBS containing 5% BSA, 2% Triton X-100, and 10% normal goat serum. Sections were incubated for 72 hours at 4°C with primary antibodies in dilution buffer. Appropriate secondary antibodies in dilution buffer were used at 1:1000, overnight at 4°C. All subsequent steps were performed in the dark and at RT. The sections were washed for 1 hour in 1× TBS and counterstained with Hoechst 33342 for 10 minutes (1:10,000; Thermo Scientific, H3570). The sections were washed for 30 minutes in 1× TBS before mounting onto slides using ProLong Gold Antifade Mountant.

### Imaging and processing.

Confocal images were acquired using a Leica TCS SP8 Confocal Laser Scanning Microscope (inverted), with the HC PL APO 40×/1.40 NA oil white objective (Leica) and HC PL APO 20×/0.75 NA dry white objective (Leica). Images were obtained under laser excitation at 407, 488, 543, and 633 nm. Emission filter bandwidths and sequential scanning acquisition were set up in order to avoid possible spectral overlap between fluorophores. Emission capture windows were set as 410–484 nm, 493–563 nm, 580–651 nm, and 670–775 nm. Pinhole was set to 65.3 μm. The confocal *Z*-stacks were processed into maximum intensity *Z*-projections using Fiji (https://imagej.net/software/fiji/). Images were labeled and assembled into a figure using Adobe Photoshop CS5 Extended (version 25.5.1×64, Adobe Systems Incorporated) and Adobe Illustrator (version 28.3, Adobe Systems Incorporated).

### Single pre-embedding peroxidase immunocytochemistry of macaque tissue.

The sections were incubated for 72 hours at 4°C with primary antibodies in TBS, and transferred for 2 hours at RT to species-specific biotinylated Fab′ or F(ab′)2 fragments in TBS. Sections were incubated with the avidin-biotin peroxidase complex (1:300; Vector Labs, PK-4000) and then visualized in 0.025% Ni-intensified 3,3-diaminobenzidine tetrahydrochloride (DAB; Sigma-Aldrich) as a chromogen in 100 mM PBS with the addition of 0.005% hydrogen peroxide for 6–12 minutes, depending on the target of interest. After the DAB reaction, sections were osmified (concentration 1%), dehydrated through a series of increasing ethanol concentrations (70%–100%), and infiltrated with propylene oxide. Tissue sections were counterstained with 1% uranyl acetate in 70% ethanol. Standard epoxy resin embedding followed typical immunoEM procedures followed by polymerization at 60°C for 48 hours. Omission of primary antibodies or substitution with nonimmune serum resulted in complete lack of immunoperoxidase labeling. Similarly, labeling was nullified when blocking the biotinylated probes with avidin/biotin.

### ImmunoEM and data analysis.

All sections were processed as described above under *Rhesus macaque brain tissue processing*. ImmunoEM imaging was conducted in LIII dlPFC (Axiophot/Axiocam HRc, Carl Zeiss). Briefly, blocks containing LIII dlPFC were sampled and mounted onto resin blocks. The specimens were cut into 50 nm sections using an ultramicrotome (Leica) and analyzed under a Talos L120C transmission electron microscope (Thermo Fisher Scientific). Several plastic blocks of each brain were examined using the 4th to 12th surface-most sections of each block (i.e., 200–600 nm), in order to sample the superficial component of sections, avoiding penetration artifacts. Structures were digitally captured at ×25,000–×100,000 magnification with a Ceta CMOS camera and individual panels were adjusted for brightness and contrast using Adobe Photoshop and Illustrator CC.2020.01 image editing software. Approximately 210 micrographs of the selected areas of neuropil with immunopositive profiles were used for analyses. For profile identification, we adopted the criteria summarized by Peters et al. (32, 89). Dendritic spines in the PFC are typically long and thin, devoid of mitochondria and with well-developed PSD. Dendritic shafts are typically round in perpendicular planes or irregularly shaped in horizontal planes, containing mitochondria, tubular, and pleomorphic cellular organelles. Axon terminals contain synaptic vesicles and the axoplasm of these terminals usually contains neurofilaments and mitochondria. The axon terminal synapses are either asymmetric, containing spherical vesicles, or symmetric, containing pleomorphic vesicles, with differences in PSD. Unmyelinated axons are small, round processes with a predominantly even and regular shape, traversing the neuropil in a straight orientation, often forming bundles in perpendicular planes. Glial processes are irregular, forming contours around neuronal elements.

### Delayed alternation task.

The mice used for behavior testing were 21–22 months old at the time of testing (*n* = 7–8 per genotype, male). Animals were tested in a T-maze (15 × 21 × 3 inches) made to be appropriate for testing mice. The maze was constructed of wood and painted black, with a guillotine door separating the start box from the main stem of the maze. Testing occurred in a small room near the colony room under normal light conditions. A sink was located on the wall to the left of the maze, and a dustpan and broom on the wall to the right of the maze; the maze was maintained in the same position in the room throughout the duration of the study. All cognitive testing was performed between 8:00 am and 5:00 pm during the animals’ light cycle; each animal was tested by the same experimenter at the same time of day, every day (Monday through Friday). Before cognitive training, all animals were exposed to the food rewards (Cheerios) in their home cage. Before moving onto the task, all mice were required to eat 10 Cheerios in less than 5 minutes and pass forced alternation. In the spatial delayed alternation task, the animal is rewarded for choosing either the left or right arm on the first trial (not counted), but from then on must always choose the arm not entered on the previous trial. The choice point of the maze was wiped with alcohol to prevent olfactory cues from guiding behavior. This process results in a 5-second delay.

### Statistics.

Quantification and statistical details of experiments can be found in the figure legends. All data are given as mean ± SEM. Nonparametric Mann-Whitney *U* test and Spearman’s correlations were conducted, as sample sizes were too small to assume normal distribution. One-way ANOVA testing with post hoc Tukey’s test was performed. Statistical significance was set to a *P* value of less than 0.05.

### Study approval.

All research was approved by the Yale Institutional Animal Care and Use Committee (IACUC) and performed under NIH *Guide for the Care and Use of Laboratory Animals* (National Academies Press, 2011).

### Data availability.

Values for all data points in graphs are reported in the Supporting Data Values file. The MS proteomics data have been deposited in the ProteomeXchange Consortium via the PRIDE partner repository with the dataset identifier PXD053288 (90).

## Author contributions

EW, AFTA, and LHS designed the research studies. EW, DD, SL, SB, HB, PC, AKA, TTL, JK, QSG, JL, EA, ALH, DC, and SU acquired data. EW, AFTA, LHS, EAJ, MKJ, JD, and DD analyzed data. PL, EAJ, ACN, and TLH provided reagents. EW, AFTA, and LHS wrote the manuscript. All authors read and approved the final manuscript.

## Funding support

This work is the result of NIH funding, in whole, and is subject to the NIH Public Access Policy. Through acceptance of this federal funding, the NIH has been given a right to make the work publicly available in PubMed Central.

- NIH grants 1F30AG074629-03 and 1T32GM136651-01 (to EW).
- NIH grant R01 AG061190-01 (to AFTA).
- NIH grants S10OD02365101A1, S10OD019967, and S10OD018034 (to the Keck MS & Proteomics Resource at the Yale School of Medicine).

## Supplementary Material

Supplemental data

Supplemental data set 1

## Figures and Tables

**Table 1:**
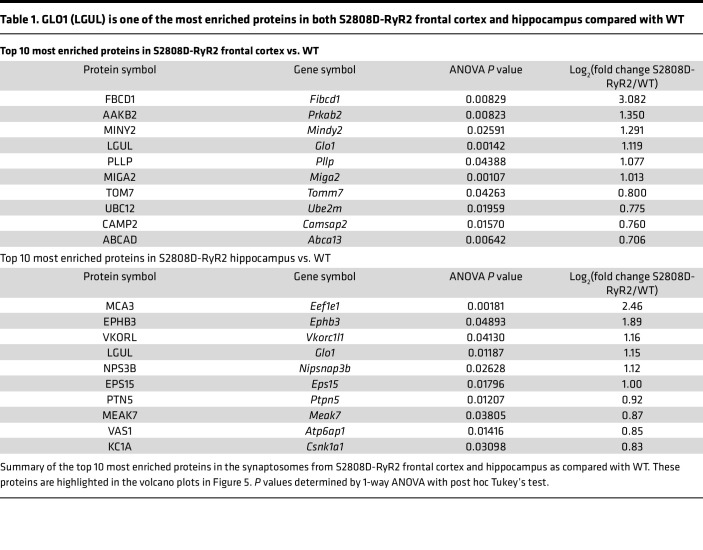
GLO1 (LGUL) is one of the most enriched proteins in both S2808D-RyR2 frontal cortex and hippocampus compared with WT

## Version 1 10/16/2025

In-Press Preview

## Version 2 11/24/2025

Electronic publication
