# Supplementary material for: Ryanodine receptor 2–mediated calcium leak is associated with increased glyoxalase I in the aging brain
Source: JCI Insight. 2025 Oct 16;10(22):e184041. doi: 10.1172/jci.insight.184041 (PMC12643534; doi:10.1172/jci.insight.184041)
Supplement: Supplemental data [file jciinsight-10-184041-s250.pdf]

**Supplemental Files.**

File S1. Proteomics raw data

Table S1. Top 10 significantly lowest enriched proteins in S2808D-RyR2 frontal cortex and hippocampus.

Table S2. Calcium signaling and related pathways are significantly increased in S2808D-RyR2 mice.

Figure S1. GLO1 expression is increased in aged entorhinal cortex of macaques.

Figure S2. GLO1 localization in the aged macaque LIII dlPFC.

Figure S3. GLO1 ultrastructural localization in aged macaque LIII dlPFC.

Figure S4. pRyR2 ultrastructure localization in LIII dlPFC of aged macaques.

Figure S5. Ultrastructure validation of synaptosome enrichment using Syn-PER.

Figure S6. GLO1 expression is increased in 3- and 12-month-old female S2808D-RyR2 mice.

Figure S7. GLO1 expression is increased as early as 1-month-old and as old as 21-month-old in S2808D-RyR2 mice.

Figure S8. GLO1 expression is increased in total brain homogenate in 3-month-old S2808D-RyR2 mice.

Figure S9. GLO1 localizes in neurons and S100$\beta$+ astrocytes in murine medial prefrontal cortex

Figure S10. GLO1 localizes in oligodendrocytes and GFAP+ astrocytes in murine medial prefrontal cortex.

Figure S11. Microglia do not express GLO1 in murine prefrontal cortex and dentate gyrus.

Figure S12. GLO1 localizes in oligodendrocytes and S100$\beta$+ astrocytes in murine dentate gyrus.

s

| Top 10 lowest enriched proteins in S2808D-RyR2 Frontal Cortex vs. WT | | | |
|---|---|---|---|
| Protein name | Gene name | ANOVA P value | $Log_2$ (Fold Change S2808D-RyR2/WT) |
| C1QA | C1qa | 0.0009698 | -0.805597 |
| TNPO1 | Tnpo1 | 0.02985659 | -0.8111806 |
| MATK | Matk | 0.04447029 | -0.819209 |
| RL40 | Uba52 | 0.04282058 | -0.8876499 |
| DDX3Y | Ddx3y | 0.03389424 | -0.9382791 |
| FIBA | Fga | 0.04865519 | -0.9850268 |
| EPHB3 | Ephb3 | 0.03102463 | -1.0583577 |
| PDIP3 | Poldip3 | 0.00157315 | -1.2034424 |
| HBA | Hba | 0.02964929 | -1.2249938 |
| REPS1 | Reps1 | 0.02869937 | -2.1253018 |
| Top 10 lowest enriched proteins in S2808D-RyR2 Hippocampus vs. WT | | | |
| Protein name | Gene name | ANOVA P value | $Log_2$ (Fold Change S2808D-RyR2/WT) |
| IQEC2 | Iqsec2 | 0.04394527 | -0.7689449 |
| PMGE | Bpgm | 0.01476851 | -0.8028818 |
| RS27L | Rps27l | 0.02228609 | -0.9783805 |
| LMBD2 | Lmbrd2 | 0.04936765 | -1.0264633 |
| ANLN | Anln | 0.04057785 | -1.0493486 |
| FXYD7 | Fxyd7 | 0.01189062 | -1.0740071 |
| RNBP6 | Ranbp6 | 0.04034484 | -1.1297358 |
| HBB1 | Hbb-b1 | 0.03472214 | -1.2892086 |
| HBA | Hba | 0.01759981 | -1.3691793 |
| DYST | Dst | 0.02009539 | -1.7400946 |

**Table S1. Top 10 significantly lowest enriched proteins in S2808D-RyR2 frontal cortex and hippocampus.** Summary of the top 10 lowest enriched proteins in the synaptosomes from S2808D-RyR2 frontal cortex and hippocampus as compared to WT. These proteins are highlighted in the volcano plots in Figure 5.

| IPA Canonical Pathways | -Log(P-Value) | Z-score |
|---|---|---|
| Calcium Signaling | 11.6 | 0.832 |
| cAMP-mediated signaling | 9.57 | 1.016 |
| Regulation of Cellular Mechanics by Calpain Protease | 7.17 | 0.894 |
| Senescence Pathway | 4.72 | 2.94 |
| Calcium Transport I | 2.33 | 0.816 |

**Table S2. Calcium signaling and related pathways are significantly increased in S2808D-RyR2 mice.** The calcium signaling and calcium signaling pathway related pathways using the proteomic data sets as derived by Ingenuity Pathway Analysis (IPA) are shown above with the corresponding Z-scores.

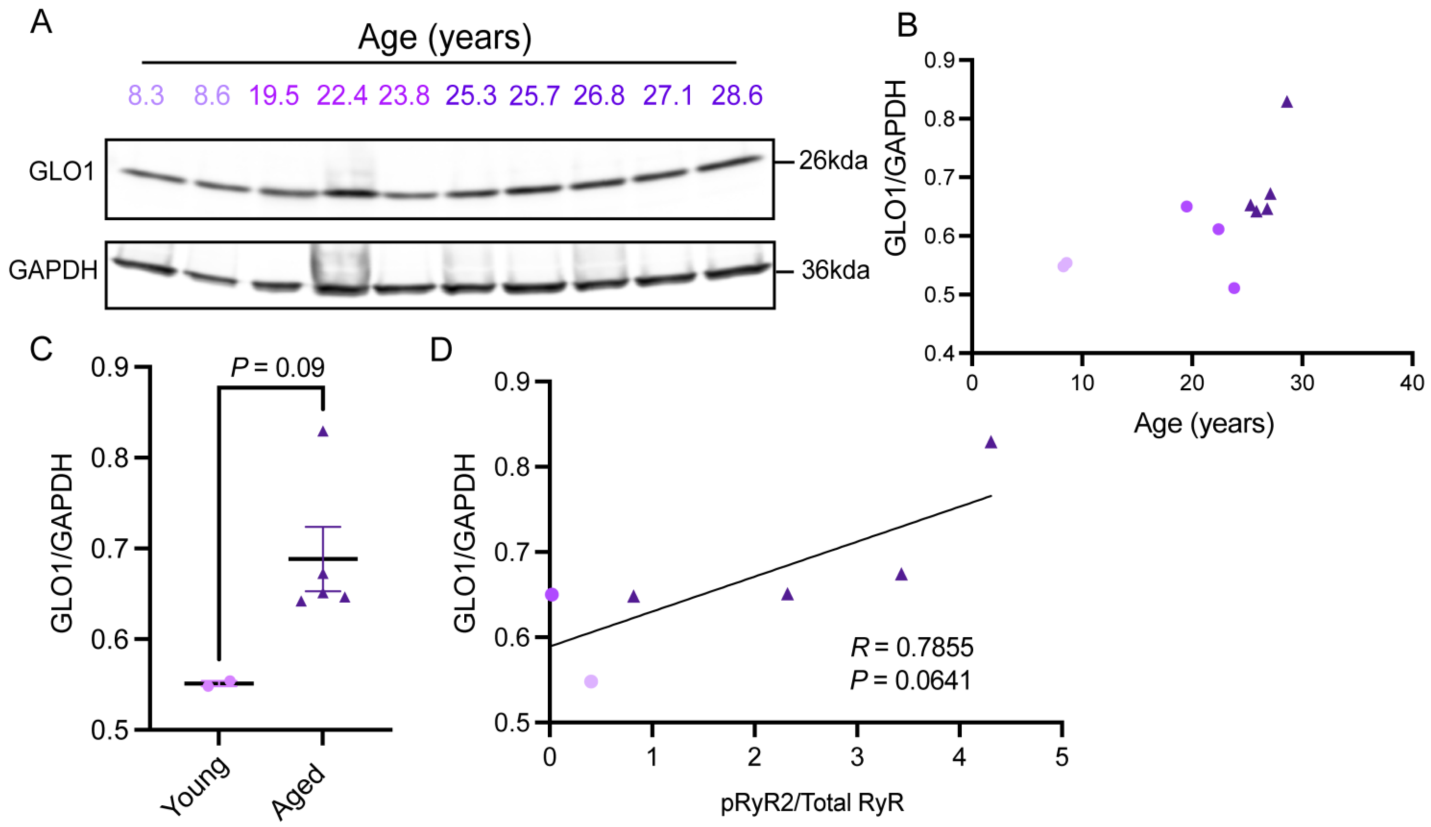


**Figure S1. GLO1 expression increases with age and is associated with pRyR2 levels in aged entorhinal cortex of macaques.** (**A**) Western blot image of GLO1 and GAPDH in macaque entorhinal cortex tissue across the age span (8.3 – 28.6 years). Age groups are color-coded (young, middle, aged). (**B**) Quantification of GLO1 increase in macaque dlPFC with age after normalization to GAPDH, plotted against age. (**C**) Quantitative analysis of GLO1 between young (<10years) and aged (≥25years). Mann-Whitney test performed. (**D**) Quantification of pS2808-RyR2 normalized by total RyR is plotted against GLO1 expression. A linear regression line with standard error is shown, with the Pearson correlation coefficient and corresponding *P* value. Data are presented as the mean ± SEM.

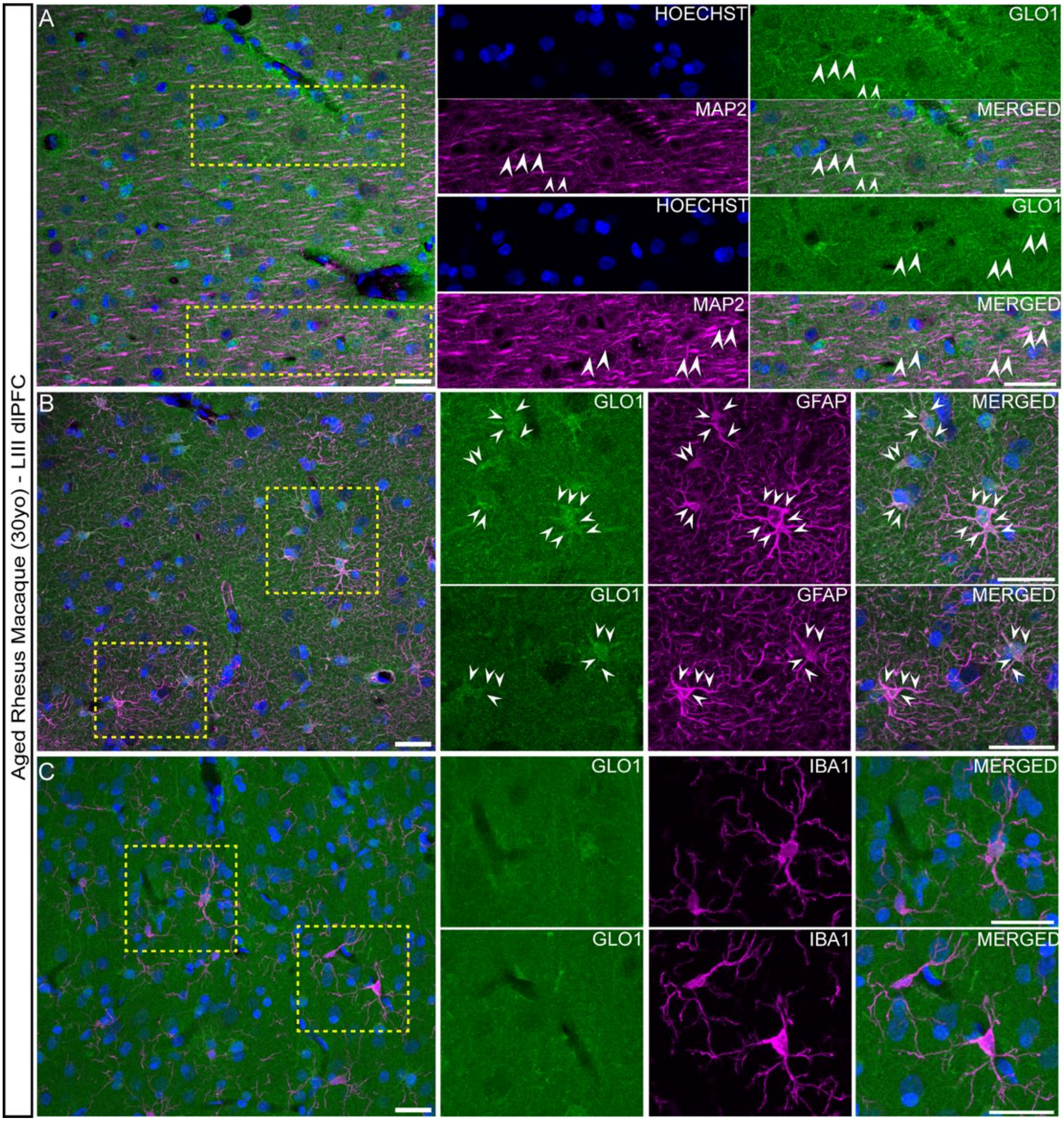


**Figure S2. GLO1 cellular localization in the aged macaque LIII dlPFC.** Representative confocal images of LIII dlPFC of an aged macaque (30 years) stained with GLO1 (green) and (**A**) excitatory neuronal markers MAP2 (magenta) and nuclei marker Hoechst (blue), (**B**) astrocytic marker GFAP (magenta) and nuclei marker Hoechst (blue), and (**C**) microglial marker IBA1 (magenta) and Hoechst (blue). The white arrowheads indicate co-localization of GLO1 within dendrites of excitatory neurons. Magnified images correspond to the dashed yellow boxes. Scale bars: 25μm.

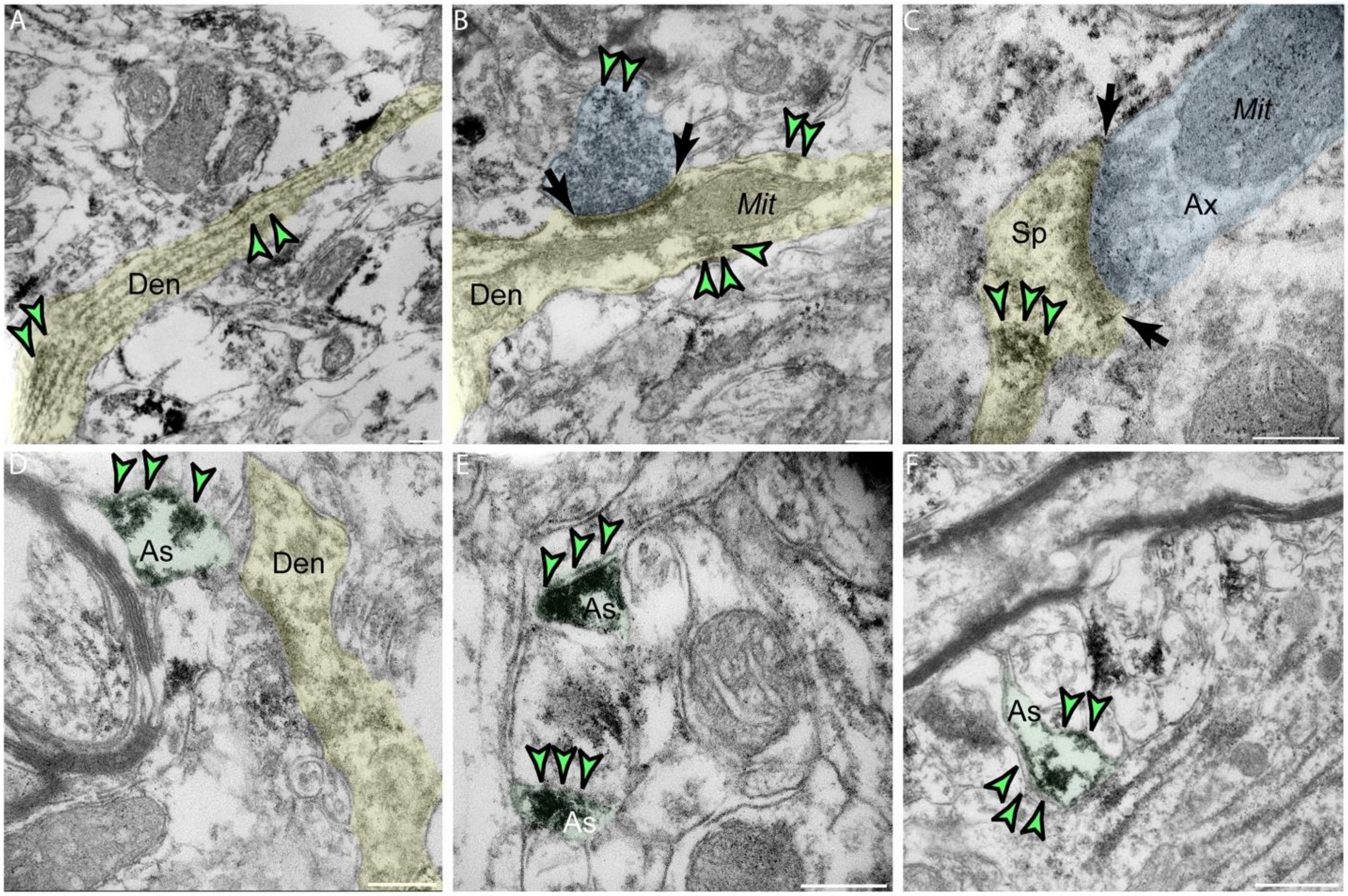


**Figure S3. GLO1 ultrastructural localization in aged macaque LIII dlPFC.** In LIII dlPFC of another aged macaque (28 years), (**A-B**) GLO1 immunoperoxidase labeling (green arrowheads) was observed along the microtubules within dendrites. (**C**) GLO1 was observed in the neck of a spine synapsing with the axon pre-terminus. GLO1 labeling was detected along the plasma membrane (**D-F**) as well as filling the glial leaflet (**E**). Profiles are pseudocolored for clarity and not all specific labeling are highlighted by arrows. *Den* dendrites (yellow), *Mit* mitochondria, *Ax* axon (blue), *Sp* spine (yellow), *As* astroglia (green). Scale bars: 500nm.

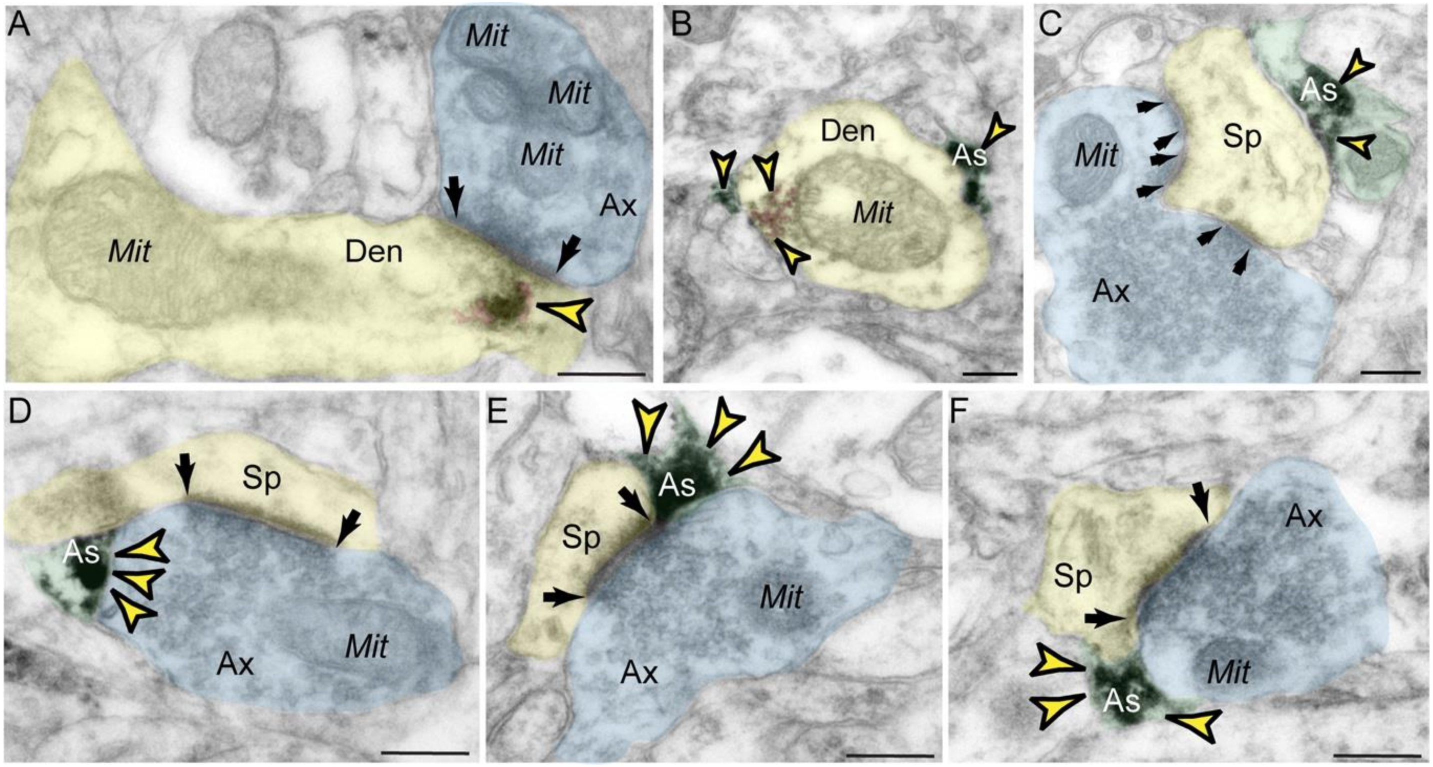


**Figure S4. pRyR2 ultrastructure localization in LIII dlPFC of aged macaques.** In aged (26 years) LIII dlPFC, pS2808-RyR2 labeling was observed in dendrites (**A-D**). Accumulation of 3,3-diaminobenzidine (DAB) labeled pS2808-RyR2 deposits (yellow arrow heads) was also observed in glia, likely astrocytic feet, that are adjacent to asymmetric synapses (**E-F**). Profiles are pseudocolored for clarity and not all specific labeling are highlighted by arrows. Synapses are between black arrows. *Den* dendrites (yellow), *Mit* mitochondria, *Ax* axon (blue), *Sp* spine (yellow), *As* astroglia (green), *SER* smooth endoplasmic reticulum (pink). Scale bars: 200nm.

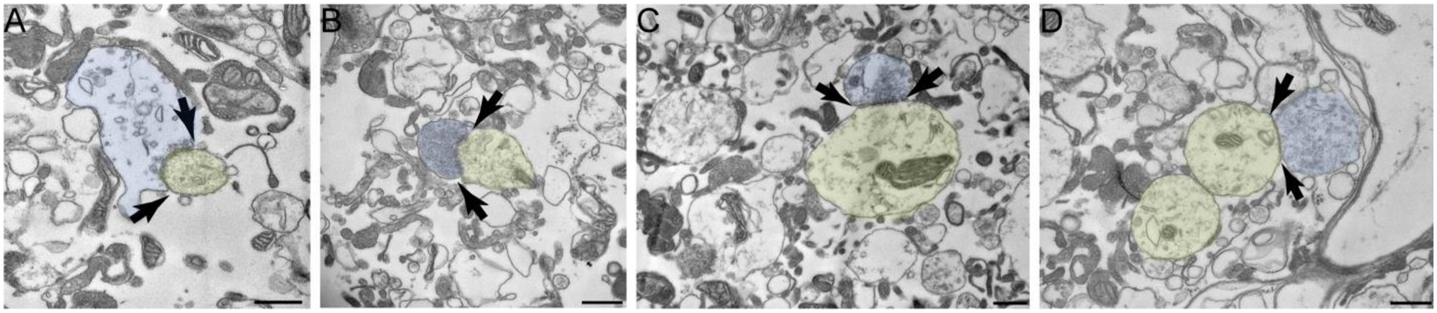


**Figure S5. Ultrastructure validation of synaptosome enrichment using Syn-PER.** Synaptosomes were processed for electron microscopy and imaged. Representative electron micrographs of synaptosomes are shown (**A-D**). Synaptosomes contained abundant SVs (**A-D**), often with mitochondrial profiles (**B-D**). Synapses are between black arrows, with the axon pre-terminus (blue) synapsing onto the post-synaptic compartment (yellow). Profiles are pseudocolored for clarity. Scale bars: 500nm.

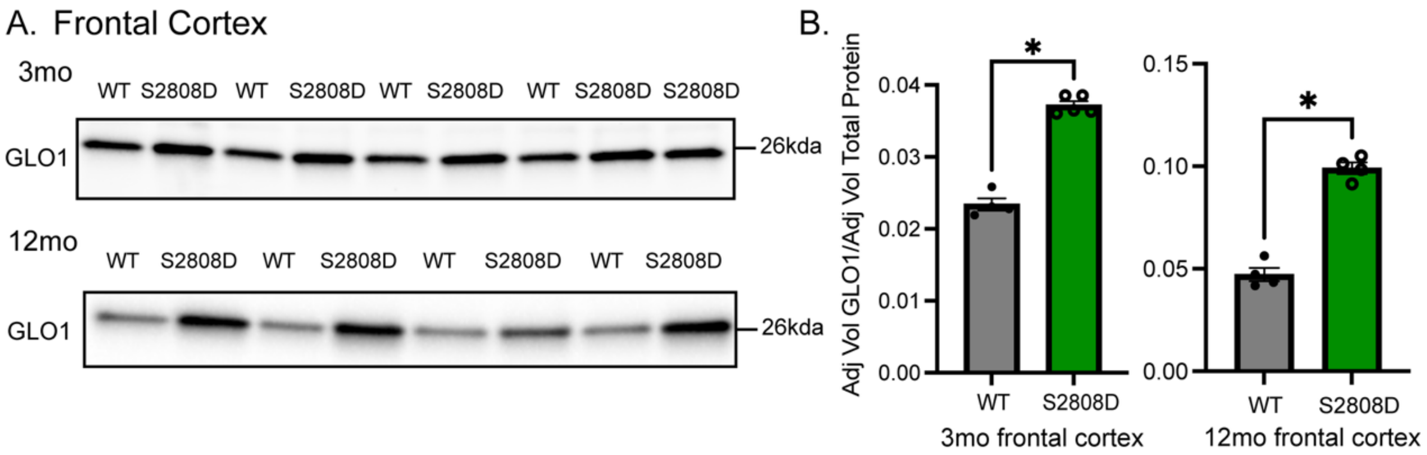


**Figure S6. GLO1 expression is increased in 3- and 12-month-old female S2808D-RyR2 mice.**

(**A**) Western blot images of GLO1 expression in synaptosomes from 3-month-old and 12-month-old female WT and S2808D-RyR2 frontal cortex. (**B**) Quantification of GLO1 expression in 3-month-old and 12-month-old normalized to total protein content based on stain free blot. n=5-8 per group per age. *P<0.05 by Mann-Whitney test.

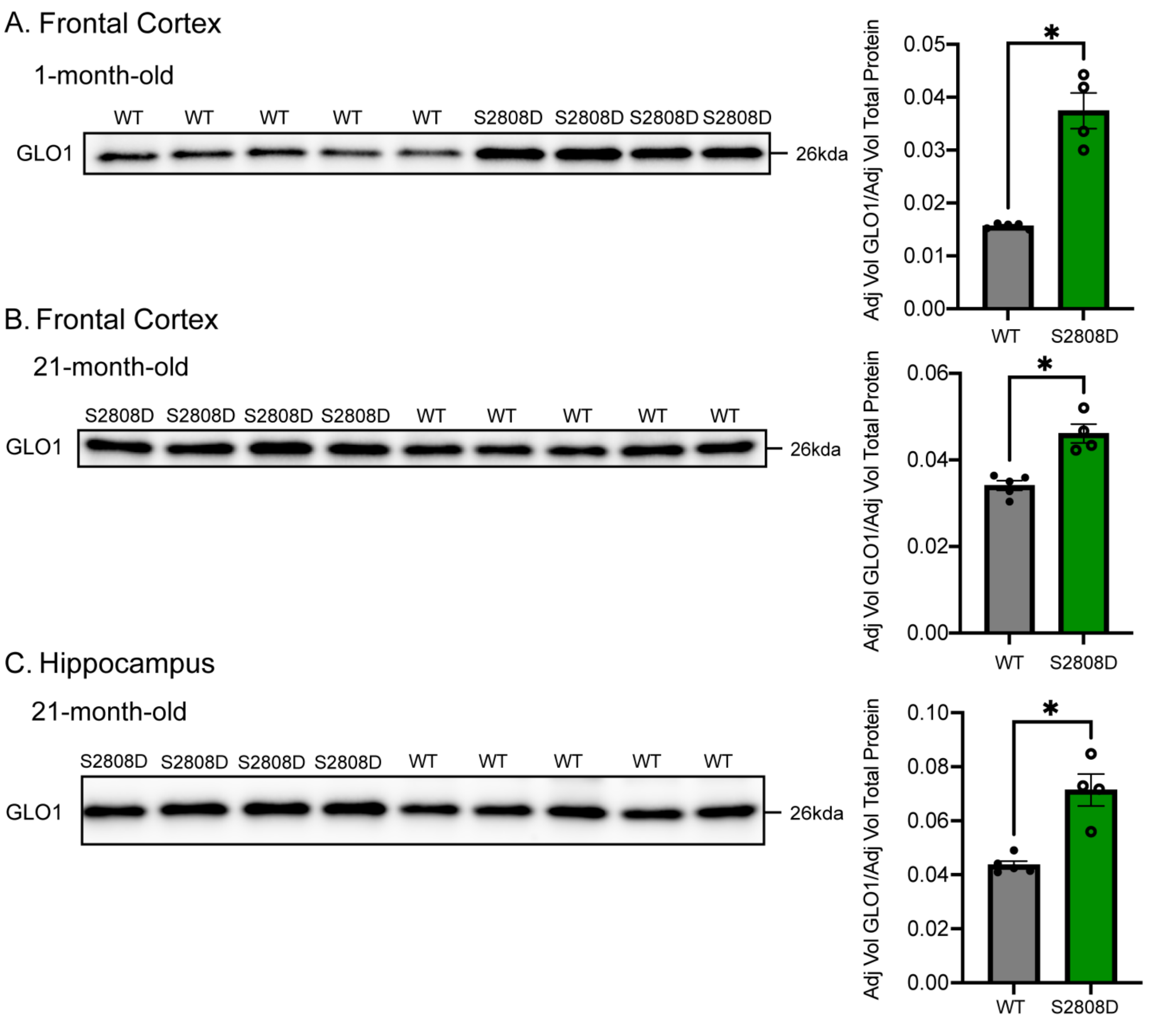


**Figure S7. GLO1 expression is increased as early as 1-month-old and as old as 21-month-old in S2808D-RyR2 mice. (A)** Western blot image of GLO1 expression in synaptosomes harvested from 1-month-old male WT and S2808D frontal cortex. GLO1 expression was normalized to total protein content based on stain free blot. Mann Whitney test. n = 4-5 per group per age. **(B)** Western blot image of GLO1 expression in synaptosomes harvested from 21-month-old male WT and S2808D frontal cortex. GLO1 expression was normalized to total protein content based on stain free blot. n = 4-5 per group per age. **(C)** Western blot image of GLO1 expression in synaptosomes harvested from 21-month-old male WT and S2808D hippocampus. GLO1 expression was normalized to total protein content based on stain free blot. n = 4-5 per group per age. *P <0.05 by Mann-Whitney test.

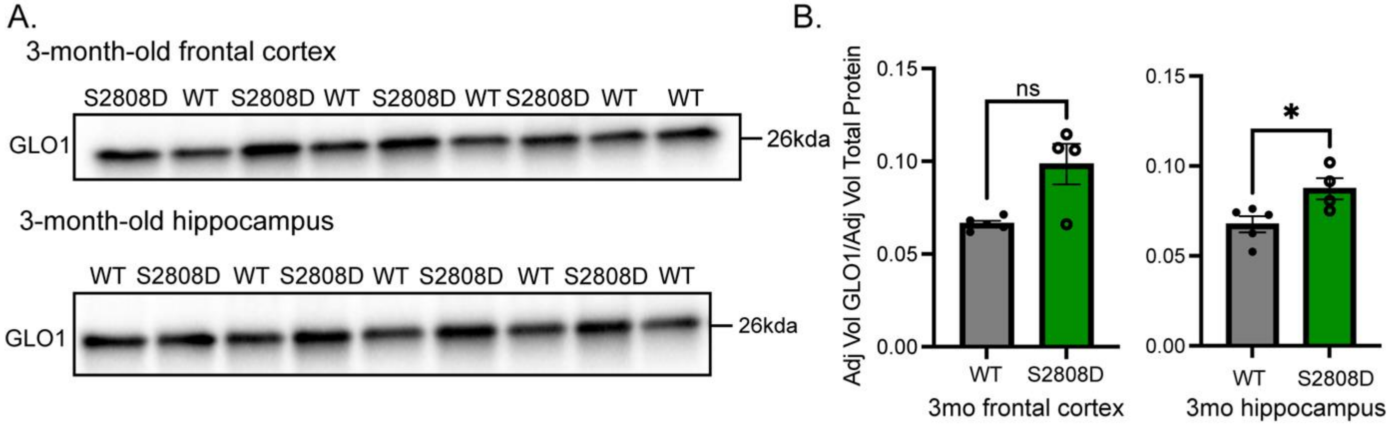


**Figure S8. GLO1 expression is increased in total brain homogenate in hippocampus of 3-month-old S2808D-RyR2 mice. (A**) Representative western blot image of GLO1 expression in total homogenate from 3-month-old male WT and S2808D-RyR2 frontal cortex and hippocampus. (**B**) Quantification of GLO1 expression normalized to total protein content based on stain free blot. n=4-5 per group per age. *P<0.05 by Mann-Whitney test.

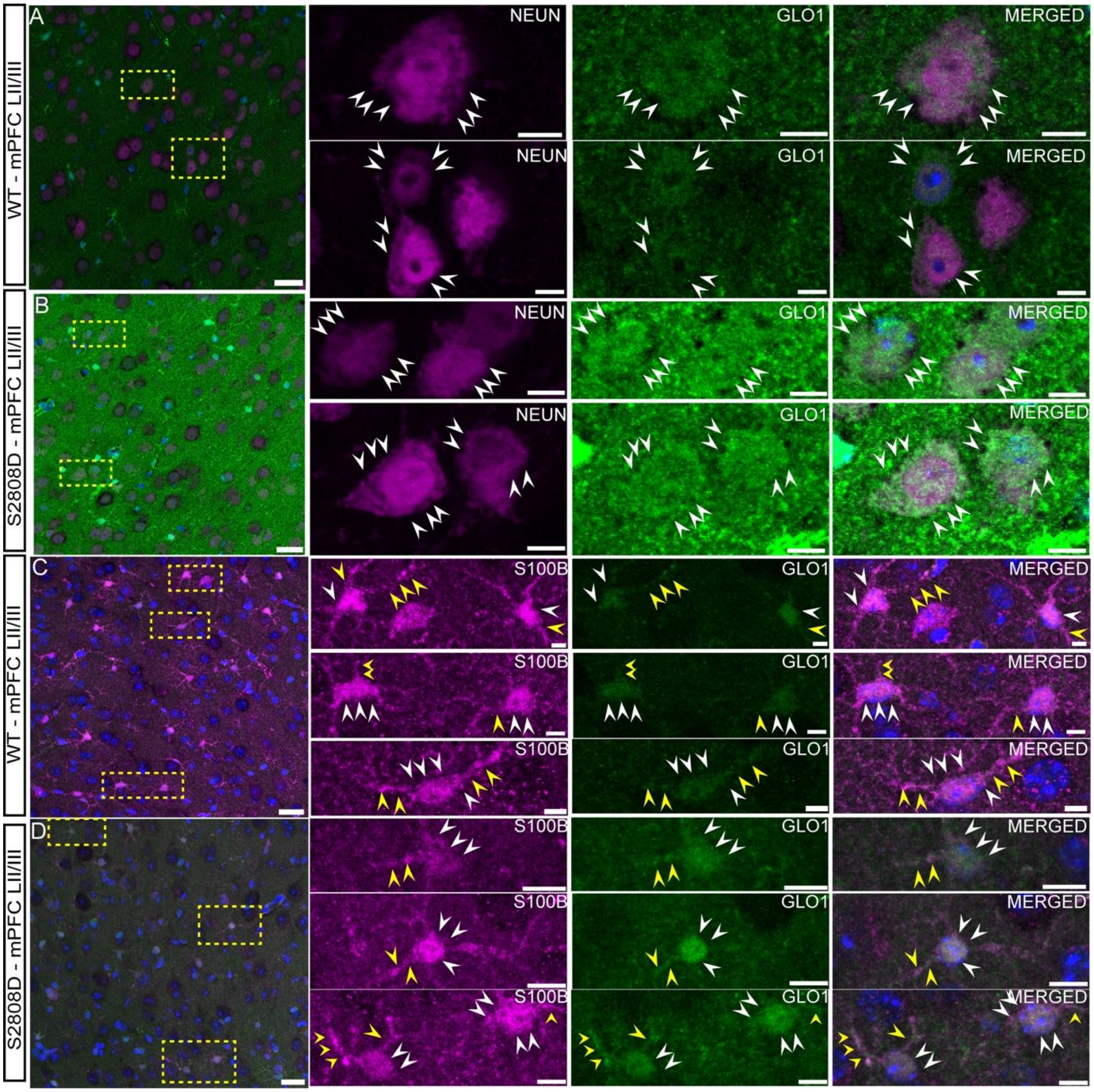


**Figure S9. GLO1 localizes in neurons and S100$\beta$+ astrocytes in murine medial prefrontal cortex.** Representative confocal images of layers II/III in the medial prefrontal cortex of 3-month-old male WT (**A and C**) and S2808D-RyR2 (**B and D**) mice. The magnified images correspond to the regions delineated by the yellow dashed boxes. (**A and B**) Neurons labeled with NeuN (magenta) colocalize with GLO1 (green), as identified by the white arrowheads. (**C and D**) Astrocytes labeled with S100$\beta$ (magenta) colocalize with GLO1 (green). Processes of astrocytes are delineated by the yellow arrowheads while the somas are indicated by the white arrowheads. *WT* wildtype, *S2808D* S2808D-RyR2. Scale bars: 25$\mu m$ (uncropped); 5$\mu m$ (cropped)

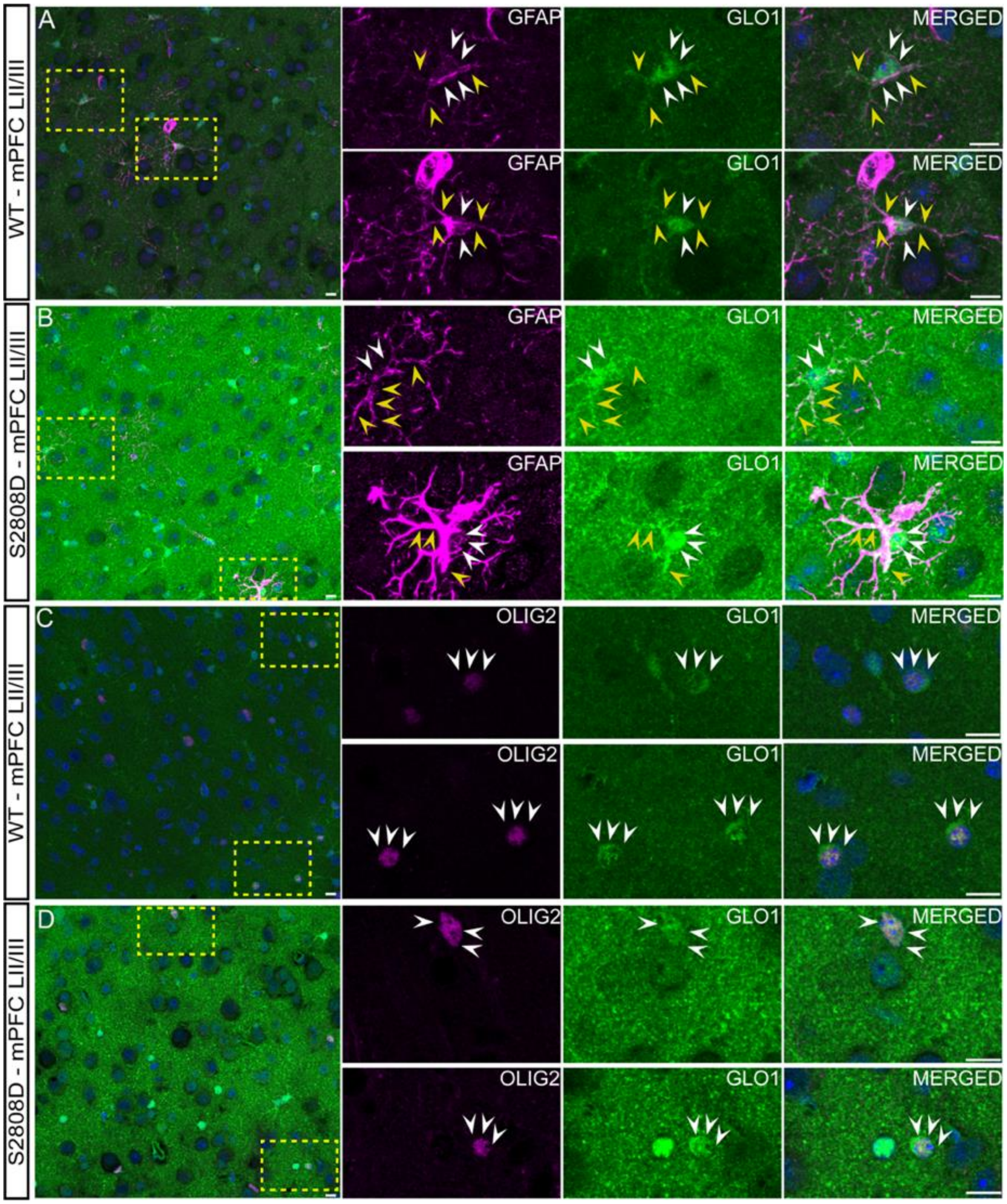


**Figure S10. GLO1 localizes in oligodendrocytes and GFAP+ astrocytes in murine medial prefrontal cortex.** Representative confocal images of layers II/III in medial prefrontal cortex of 3-month-old WT (**A and C**) and S2808D-RyR2 (**B and D**) mice. The magnified images correspond to the regions delineated by the yellow dashed boxes. (**A and B**) Astrocytes co-localized with GFAP (magenta) and GLO1 (green). (**C and D**) Oligodendrocytes and OPCs labeled with Olig2 (magenta) colocalized with GLO1 (green), as identified by the white arrowheads. Processes of astrocytes are delineated by the yellow arrowheads while the somas are indicated by the yellow arrowheads. n=3 per group. Scale bars: 10μm.

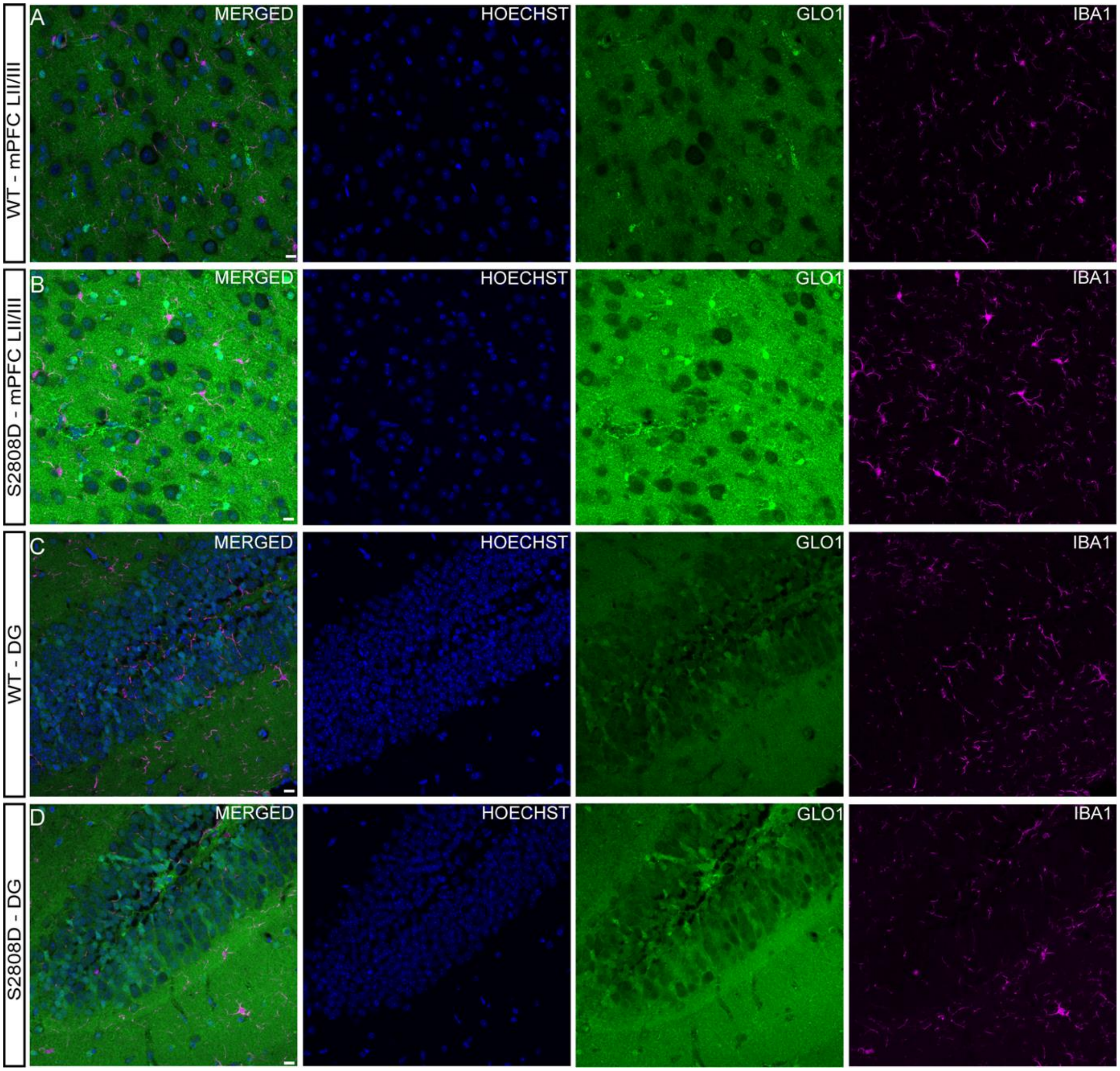


**Figure S11. Microglia do not express GLO1 in murine prefrontal cortex and dentate gyrus.** Representative confocal images of layers II/III in medial prefrontal cortex (**A-B**) and dentate gyrus (**C-D**) of 3-month-old male WT (**A and C**) and S2808D-RyR2 (**B and D**) mice. Tissue was stained with nuclear stain Hoechst (blue), GLO1 (green), and microglial marker Iba1 (magenta). No co-localization was detected between Iba1 and GLO1. n=3 per group. Scale bars: 10μm.

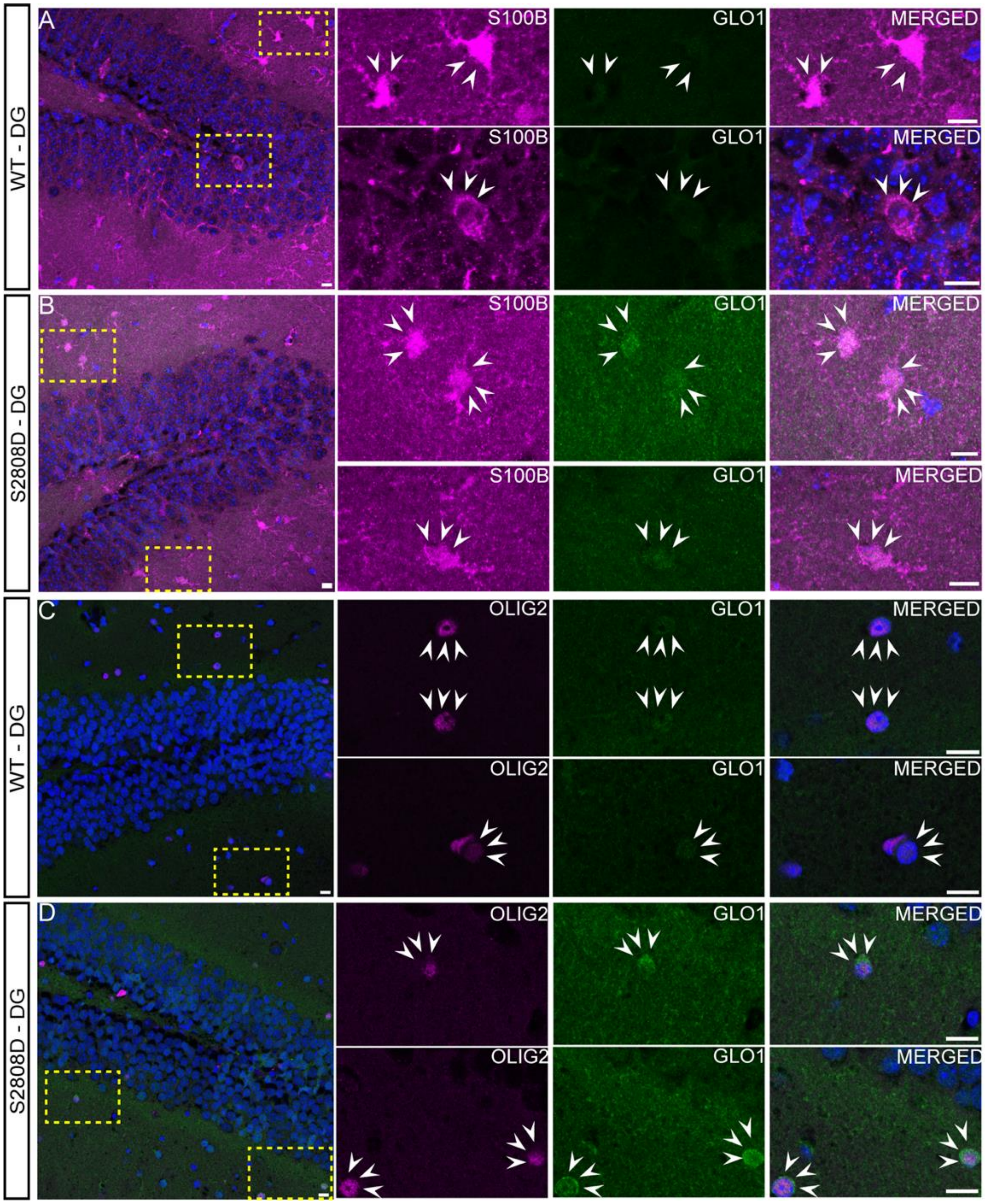


**Figure S12. GLO1 localizes in oligodendrocytes and astrocytes in murine dentate gyrus.**

Representative confocal images of layers II/III in dentate gyrus of 3-month-old WT (**A and C**) and S2808D-RyR2 (**B and D**) mice. The magnified images correspond to the regions delineated by the yellow dashed boxes. (**A and B**) Astrocytes co-localized with S100$\beta$ (magenta) and GLO1 (green). (**C and D**) Oligodendrocytes and OPCs labeled with Olig2 (magenta) colocalized with GLO1 (green), as identified by the white arrowheads. Somas of astrocytes are indicated by the white arrowheads. n=3 per group. Scale bars: 10$\mu$m.
